# *Lrrk2* G2019S mutation incites increased cell-intrinsic neutrophil effector functions and intestinal inflammation in a model of infectious colitis

**DOI:** 10.1038/s41531-025-01077-x

**Published:** 2025-08-29

**Authors:** Jessica Pei, Nathalia L. Oliveira, Sherilyn J. Recinto, Alexandra Kazanova, Celso M. Queiroz-Junior, Ziyi Li, Katalina Couto, Susan Westfall, Ahmed M. Fahmy, Camila Tiefensee-Ribeiro, Irah L. King, Austen J. Milnerwood, Michel Desjardins, Ajitha Thanabalasuriar, Jo Anne Stratton, Samantha Gruenheid

**Affiliations:** 1 Department of Microbiology and Immunology, McGill University, Montreal, QC Canada; 2 Aligning Science Across Parkinson’s (ASAP) Collaborative Research Network, Chevy Chase, MD USA; 3 Montreal Neurological Institute, McGill University, Montreal, QC Canada; 4 Departamento de Morfologia, Universidade Federal de Minas Gerais, Belo Horizonte, Brazil; 5 Department of Pharmacology and Therapeutics, McGill University, Montreal, QC Canada; 6 Département de pathologie et biologie cellulaire, Université de Montréal, Montreal, QC Canada

**Keywords:** Inflammation, Microbiology

## Abstract

Parkinson’s Disease (PD) is a neurodegenerative disorder often preceded by gastrointestinal dysfunction. Mutations in leucine-rich repeat kinase 2 (*LRRK2*) are known risk factors for both PD and inflammatory bowel disease (IBD), suggesting a link between PD and the gastrointestinal tract. Using single-cell RNA-sequencing and spectral flow cytometry, we demonstrated that the *Lrrk2* Gly2019Ser (G2019S) mutation is associated with an increased neutrophil presence in the colonic lamina propria during *Citrobacter rodentium* infection. This concurred with a Th17 skewing, upregulated *Il17a*, and greater colonic pathology during infection. In vitro experiments showed enhanced kinase-dependent neutrophil chemotaxis and neutrophil extracellular trap (NET) formation in *Lrrk2* G2019S mice compared to wild-type counterparts. Our results add to the understanding of LRRK2-driven immune cell dysregulation and its contribution to PD, offering insights into potential biomarkers for early diagnosis and intervention in PD.

## Introduction

Parkinson’s disease (PD) is a neurological disease associated with aging. It is the second most prevalent neurodegenerative disorder after Alzheimer’s disease and is predicted to affect 14 million individuals by 2040^1^. Hallmark symptoms of PD include bradykinesia, rigidity, and resting tremor, which are linked to the loss of dopaminergic neurons in the substantia nigra pars compacta region of the brain^2^. The average age of PD diagnosis is about 60 years of age, when it is estimated that ~50% of dopaminergic neurons have already been destroyed^3^. Currently accessible treatments can alleviate some PD motor symptoms, but these therapies lose effectiveness over time and do not target the wide range of other symptoms^4^. PD is associated with a diverse range of non-motor symptoms that significantly increase the overall burden of the condition. This includes sleep disturbances, hyposmia, and gastrointestinal issues such as constipation, dysphagia, and small intestinal bacterial overgrowth, which can manifest decades before the disease progresses to motor dysfunction^5,6^. There is therefore considerable interest in the field to increase our understanding of the early, pre-motor pathophysiology of PD, essential for better early diagnosis and management of the disease.

Of note, several studies have indicated that PD is more common in people with inflammatory bowel disease (IBD) and those with previous evidence of intestinal infection^7,8^, suggesting that intestinal inflammation can contribute to PD pathogenesis. Mutations in the leucine-rich repeat kinase 2 gene (*LRRK2*) are responsible for ~1% of all PD cases and also confer increased risk for IBD^9,10^. Among them, LRRK2 Gly2019Ser (G2019S), located within the kinase domain, is the most common PD-associated mutation and increases kinase activity^11–13^. In contrast to the brain, *LRRK2* is extensively expressed in peripheral organs such as the lungs, spleen, and kidneys^14,15^. *LRRK2* is highly expressed in a variety of immune cells, including neutrophils, macrophages, monocytes, and B cells^16–18^, suggesting a possible immune mediated effect of *LRRK2* mutation. Indeed, studies have shown that LRRK2 may play a role in controlling inflammation and pathogen defense in bacterial diseases. LRRK2 has been implicated in susceptibility to *Listeria monocytogenes*^19^ and in the control of *Salmonella* Typhimurium^20,21^. Fang et al. recently demonstrated that male mice with bacterial artificial chromosome (BAC)-transgene overexpression of human *LRRK2* G2019S showed increased severity of intestinal inflammation following dextran sodium sulfate (DSS)-induced colitis, along with changes in the severity of PD-related neuropathology and behaviour^22^. Others have found a relationship between *Lrrk2* mutations and the induction of type I IFN gene expression^23^.

Here, we sought to systematically investigate the effects of the PD causal *Lrrk2* G2019S mutation in the mouse endogenous genome (G2019S knock-in mice) at steady state and in the early response to intestinal infection. Using the *Citrobacter rodentium* model of self-limiting infectious colitis, we show that LRRK2 G2019S promotes increased colon immunopathology following infection, which is associated with an influx of neutrophils and differential gene regulation in immune cells, including monocytes and neutrophils. Furthermore, we demonstrate a cell-intrinsic role for LRRK2 G2019S in increasing neutrophil migration and neutrophil extracellular trap (NET) formation. We also observed an upregulation of Th17 immune responses, which may together contribute to the observed increased colon pathology. Collectively, these findings increase our understanding of the role of PD-associated genes in immune cells and their contribution to immune dysregulation, which could contribute to the development of pharmacological targets and biomarkers for early detection and intervention in PD.

## Results

### *Lrrk2* G2019S mutation does not affect *C. rodentium* colonization or clearance

*C. rodentium* is a gram-negative natural intestinal pathogen of mice that forms characteristic attaching and effacing (A/E) lesions to adhere to the intestinal mucosa and cause self-limiting colitis in most inbred mouse strains. This mechanism is conserved among the human pathogens enteropathogenic *Escherichia coli* (EPEC) and enterohaemorrhagic *E. coli* (EHEC). *C. rodentium* shares 67% of its genes with both EHEC and EPEC, including genes encoding virulence factors such as a type III secretion system and several of its associated effector proteins that are injected into host cells. *C. rodentium* infection of mice is therefore widely used as a model system to study pathogenesis and host responses to gram-negative bacteria-induced intestinal inflammation^24^.

**Fig. 1:**
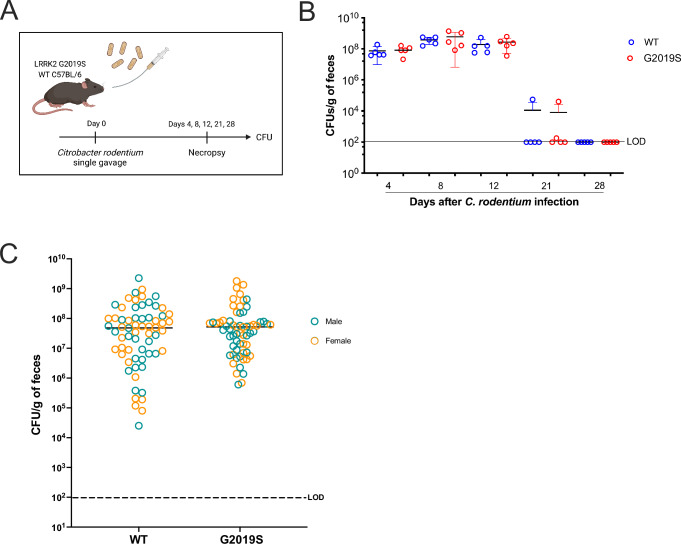
*Lrrk2* G2019S mutation does not interfere with *C. rodentium* colonization and clearance. **A** Experimental design. Male and female *Lrrk2* G2019S and WT mice (male-to-female ratio = 0.67) were gavaged once with ~1 × 10^9^ colony-forming units (CFUs) of *C. rodentium*. Bacterial fecal shedding was quantified on days 4, 7, or 8, 12, 21, and 28 after infection. **B** CFUs per g of feces. Data are represented as mean ± SD and analyzed by two-way ANOVA with Sidak post-test. One representative of two independent experiments is presented. *n* = 5 mice per group. **C** Comparison fecal CFU *C. rodentium* burden in WT and G2019S mice on day 7 of infection, separated by sex. Data are represented as mean ± SD and analyzed by t-test. Ten independent experiments are presented. *n* = 64–65 mice per group. Dashed lines indicate the limit of detection (LOD).

We sought to investigate whether the PD-associated variant, *Lrrk2* G2019S, influenced the host response to this natural mouse pathogen. To gain insight into the role of *Lrrk2* G2019S in the control of *C. rodentium* infection, *Lrrk2* G2019S knock-in and wild-type (WT) mice were infected with *C. rodentium* in a single gavage with ~1 × 10^9^ colony-forming units (CFU). Bacterial colonization was evaluated at specific time points following the infection using fecal *C. rodentium* burden as a surrogate readout (Fig. 1A). By day 4 post-infection (p.i.), mice already presented with high levels of *C. rodentium*, indicating that the infection was well established in both genotypes. By day 8 p.i., mice reached the peak of bacterial load, which decreased slightly by day 12. On day 21, bacterial clearance was progressing in both genotypes, with the majority of mice reaching the limit of CFU detection (LOD). On day 28, all the mice had cleared the infection. Overall, no differences in bacterial colonization or clearance were observed between WT and *Lrrk2* G2019S mice during the infection (Fig. 1B). Since previous work has indicated a sex difference in some LRRK2-associated phenotypes, we also assessed multiple cohorts of mice for *C. rodentium* loads at day 7 p.i., the peak of bacterial load, and compared sex differences in bacterial clearance. Female and male mice from both genotypes had similar burden of *C. rodentium* on day 7 p.i. (Fig. 1C).

To further explore the impact of the G2019S mutation in LRRK2 during *C. rodentium* infection, we evaluated the overall response of these mice in early infection. We found that mice weight variation was similar among uninfected and infected groups and between different genotypes. Moreover, no differences in weight loss by sex were seen (Supplementary Fig. 1A). Gut motility was also evaluated by counting the number of fecal pellets excreted per hour, after 5 days of infection. Although there is a trend towards increased fecal pellets per hour after infection of WT mice, no major differences were detected between the genotypes of infected mice (Supplementary Fig. 1B). Moreover, on day 7 of infection, fecal water content was similar in uninfected groups and tended to decrease after infection, reaching statistical significance only for G2019S mice (Supplementary Fig. 1C). Thus, indicating that *Lrrk2* G2019S mice have similar control of *C. rodentium* infection, with minor changes in fecal water composition.

### Single-cell RNA sequencing (scRNAseq) and flow cytometry show increased neutrophil presence in *Lrrk2* G2019S mice following infection

**Fig. 2:**
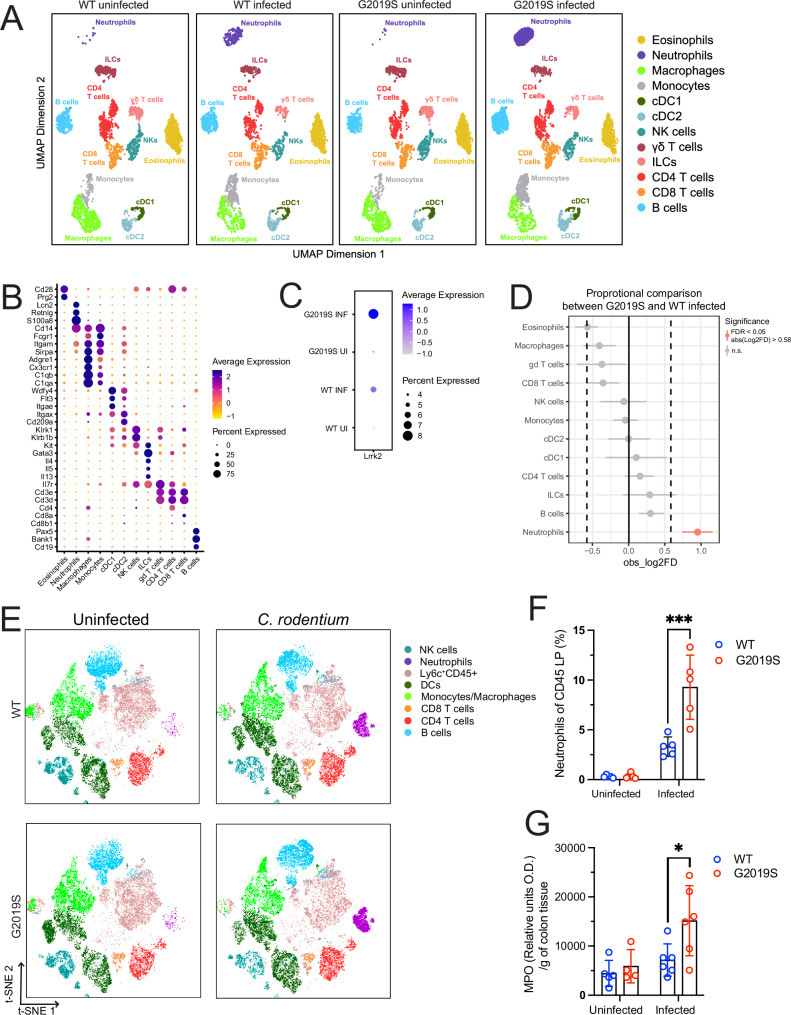
Single cell RNA sequencing (scRNAseq) and flow cytometry show increased neutrophil presence in colonic lamina propria of *Lrrk2* G2019S mice following infection. Male and female *Lrrk2* G2019S and WT mice were gavaged once with ~1 × 10^9^ CFUs of *C. rodentium,* and colons were harvested. Immune cells were isolated for scRNAseq and flow cytometry. **A** Unsupervised clustering of immune cells in WT or G2019S uninfected and infected mice. The scRNAseq datatset contains 13,892 cells total sequenced at a depth of ~20,000 genes per cell, depicted on respective UMAP plots. **B** Dotplot depicting identifying markers used to annotate each immune cluster based off literature. **C** Dotplot depicting average *Lrrk2* expression across all immune cell clusters, split by condition. **D** Proportional analysis conducted through permutation test comparing G2019S-infected and WT-infected conditions. Significance cut off: FDR < 0.05. scRNAseq was obtained from pooled conditions of *n* = 3 mice per group (male-to-female ratio = 0.5). One independent experiment is presented. **E** Flow cytometry results of immune cell clusters projected onto t-SNE plots. **F** Percent Ly6G+ neutrophils isolated from CD45+ immune cells of the colonic lamina propria. Data are represented as mean ± SD and analyzed by two-way ANOVA with Fisher’s LSD post-test. *n* = 5–6 mice per group (male-to-female ratio = 0.9). ***p < 0.001. **G** MPO enzymatic assay results of colonic tissue sample measured through O.D. and normalized over gram of colonic tissue. Data are represented as mean ± SD and analyzed by two-way ANOVA with Fisher’s LSD post-test. *n* = 4-6 mice per group (male-to-female ratio = 0.42). *p < 0.05. One representative of two independent experiments is presented.

While *LRRK2* is known to be highly expressed in immune cells upon stimulation^25^, its role in regulating inflammation and infection in the intestine remains unclear^26,27^. To understand how *Lrrk2* G2019S mutation affects the immunophenotype of the colonic lamina propria at baseline and during infection, we completed scRNAseq. Colons were harvested from WT and *Lrrk2* G2019S mice at 7 days p.i., a timepoint when innate immune changes are prominent in response to *C. rodentium* infection^24^. In parallel, colons were also collected from age-and sex-matched uninfected WT and *Lrrk2* G2019S controls. Using *Seurat*, we integrated datasets from our four conditions, following standard quality control metrics (number of unique molecular identifiers (UMIs) per cell, percent mitochondrial reads)^28^. Post filtering, we yielded a total of 13,892 cells sequenced at a depth of ~20,000 genes per cell, depicted on respective UMAP plots (Fig. 2A). Through unsupervised clustering and manual annotation based on established transcriptional markers for immunocytes within the lamina propria, all major immune cell types were identified^29,30^ (Fig. 2B). Although at baseline there were no noticeable differences in cluster sizes between genotypes, the data from infected mice indicated increased representation of neutrophils in both genotypes, with a greater effect in *Lrrk2* G2019S than WT (Fig. 2A). Across the four conditions, *Lrrk2* expression was low to moderate (4–8% of total immunocytes) which is consistent with other published datasets, showing that detection of *Lrrk2* mRNA and protein can be challenging^14,17^. *Lrrk2* G2019S-infected immunocytes showed higher average *Lrrk2* expression relative to all other conditions (Fig. 2C). *Lrrk2* expression was highest in B cells and cDCs (Supplementary Fig. 2A, B), consistent with previous findings^17^.

To further understand how LRRK2 G2019S activity may alter immunocyte proportions in the lamina propria under infectious conditions, we completed a proportional analysis of *Lrrk2* G2019S against WT infection. Confirming what was observed in Fig. 2A, neutrophils were the only cluster with a significant proportional upregulation in *Lrrk2* G2019S-infected mice (Fig. 2D). To validate our scRNAseq findings, we immunophenotyped the colonic lamina propria of WT and *Lrrk2* G2019S mice at baseline and day 7 p.i. through flow cytometry. Consistent with our scRNAseq data, t-SNE pseudo dot plots of lamina propria cells showed no distinguishable differences between cluster sizes of uninfected mice across genotypes, but a significant neutrophil increase in infected G2019S mice compared to WT (Fig. 2E, F, Supplementary Fig. 3). To further investigate neutrophil presence in the lamina propria, immunofluorescent staining and microscopy were performed. We observed that neutrophil presence increased after infection, particularly at the crypt base (Supplementary Fig. 4). The increase in neutrophils was confirmed by the activity of myeloperoxidase (MPO), an indirect measure of neutrophil presence^31^. As expected, G2019S-infected mice showed higher levels of MPO activity compared to WT-infected (Fig. 2G). Together, our findings demonstrate a marked increase in neutrophils in the colonic lamina propria of infected *Lrrk2* G2019S mice, compared to similarly infected WT littermate mice.

### Differentially expressed gene analysis indicates neutrophils of *Lrrk2* G2019S-infected mice have an increased pro-inflammatory profile

**Fig. 3:**
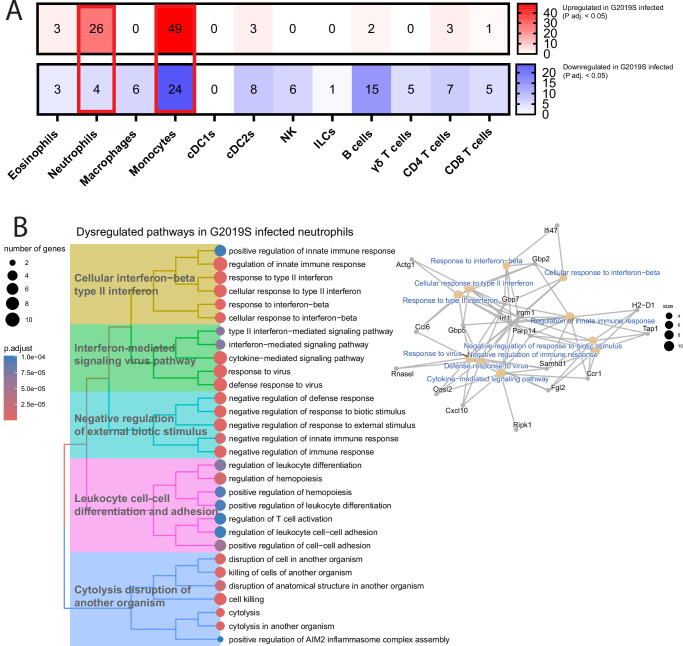
Differentially expressed gene analysis indicates neutrophils of *Lrrk2* G2019S-infected mice have an increased pro-inflammatory profile. Male and female *Lrrk2* G2019S and WT mice (male-to-female ratio = 0.5) were gavaged once with ~1 × 10^9^ CFUs of *C. rodentium*. **A** Differential gene expression (DEG) analysis comparing G2019S and WT-infected mice for each immune cell cluster calculated using Seurat differential expression analysis by non-parametric Wilcoxon rank sum test with Bonferroni correction. Adjusted P value cut off = 0.05, Q value cut off = 0.2. Upregulated genes are depicted as red in the heatmap, and downregulated genes are depicted in blue. **B** Pathway enrichment analysis completed on DEGs obtained by comparing G2019S and WT-infected neutrophils. DEGs were run against Gene ontology (GO) biological pathways database using *ClusterProfiler* V4.12.6 (RRID:SCR_016884). P value cut off = 0.05, Q value cut off = 0.2. Top 30 GO term categories are depicted as a Treeplot (Left). Cnetplots depict association of differentially expressed gene hits, and which GO term pathways they correspond to (Right). scRNAseq was obtained from pooled conditions of *n* = 3 mice per group. One independent experiment is presented.

To understand how the *Lrrk2* G2019S mutation affects gene regulation, differential gene expression analysis was performed on each immune cell cluster, comparing WT and *Lrrk2* G2019S-infected mice. The number of significant up and downregulated gene hits for each cluster are represented in a heatmap (Fig. 3A). Among all the clusters, neutrophils and monocytes had the highest number of differentially expressed genes (DEGs). In neutrophils from infected *Lrrk2* G2019S mice, there were 26 significantly upregulated genes, and 4 significantly downregulated genes, compared to infected WT mice (Tables 1 and 2). Several upregulated genes of interest include neutrophil migration and maturation markers *Plaur, Fgl2*, chemokines *Cxcl10, Ccl6, Ccr1*, and interferon (IFN) signaling-related genes *Irf1* and *Irgm1*^32^. Using our total DEG list, we conducted pathway enrichment analysis through Gene Ontological (GO) terms to identify key associated biological pathways. GO terms pointed to pro-inflammatory signaling, including increased IFN-mediated signaling (type II IFN-mediated signaling, defense/response to virus, and cytokine-mediated signaling), cellular response to IFN-β, and cytolysis disruption of another organism (defense response to bacterium and cell killing) (Fig. 3B).

**Table 1:** Top significantly upregulated differentially expressed genes in G2019S infected neutrophils compared to WT infected

Genes	P value	Average Log2FC	Percent 1	Percent 2	Adjusted P values
Plaur	5.7334E-18	1.19644481	0.722	0.466	1.1929E-13
Irf1	7.3878E-13	0.7562717	0.79	0.561	1.5371E-08
Actg1	6.2192E-11	0.48975743	0.982	0.951	1.294E-06
Oasl2	2.5611E-10	0.74876679	0.577	0.341	5.3286E-06
Cxcl10	2.7398E-09	1.22761205	0.491	0.269	5.7004E-05
Fgl2	3.934E-09	0.74520803	0.757	0.587	8.1851E-05
Tap1	5.7094E-09	0.64319616	0.572	0.323	0.00011879
Irgm1	9.4406E-09	0.72263765	0.489	0.256	0.00019642
Samhd1	1.4767E-08	0.4612426	0.888	0.776	0.00030725
Sdcbp	3.4171E-08	0.53264354	0.845	0.691	0.00071097
Rhoh	3.7982E-08	0.71607469	0.402	0.202	0.00079026
Slfn5	1.233E-07	1.1046694	0.347	0.166	0.00256543
Retnlg	2.0814E-07	0.73451243	0.673	0.484	0.0043306
Srgn	2.7072E-07	0.25254382	0.998	1	0.00563266
Ccl6	3.9694E-07	0.66280572	0.683	0.48	0.00825871
Gbp5	5.5947E-07	0.73392072	0.593	0.408	0.0116403
Rnasel	5.7304E-07	0.74939407	0.375	0.188	0.01192274
H2-D1	8.0097E-07	0.30977592	0.985	0.951	0.01666495
Nampt	8.9611E-07	0.58221739	0.579	0.381	0.01864439
Gbp7	1.0069E-06	0.50232508	0.527	0.318	0.02094937
Parp14	1.2949E-06	0.46035841	0.595	0.39	0.02694095
Gbp2	1.5309E-06	0.66079582	0.65	0.462	0.03185193
Ccr1	1.5835E-06	0.46078703	0.86	0.74	0.03294537
Gdap10	1.7481E-06	0.66998806	0.36	0.184	0.03637173
Ripk1	2.0795E-06	0.82825161	0.316	0.161	0.04326531
Ifi47	2.2705E-06	0.5760027	0.428	0.238	0.0472395

**Table 2:** Top downregulated differentially expressed genes in G2019S infected neutrophils compared to WT infected

Genes	P value	Average Log2FC	Percent 1	Percent 2	Adjusted P values
Ighg1	6.5242E-20	−1.0026766	0.055	0.283	1.3574E-15
Igha	2.9059E-15	−0.5294435	0.987	1	6.0461E-11
Igkc	4.9902E-09	−0.3962442	0.995	0.996	0.00010383
Malt1	2.3045E-06	−0.6450959	0.511	0.65	0.04794689

Monocytes from infected *Lrrk2* G2019S mice showed 49 upregulated genes and 24 downregulated genes, which are depicted in Supplementary Tables 1 and 2. GO term analysis indicated dysregulation in cellular response to infection and presentation of peptides via MHC II (Supplementary Fig. 5A). Specifically, changes in MHC II peptide presentation were driven by downregulation of *H2-Aa* and *H2-Ab1*, which encode α and β components of the MHC II complex, respectively. In addition to gene expression differences, flow cytometry revealed increased influx of Ly6c^High^ monocytes (Supplementary Fig. 5B). This was further supported through sub-clustering of monocytes in the scRNAseq dataset into Ly6c2^Low^ or Ly6c2^High^, indicating mature or immature monocytes, respectively (Supplementary Fig. 5C). Index analysis showed that Ly6c2^High^ monocytes followed similar trends in scRNAseq compared to flow cytometry data (Supplementary Fig. 5D). Taking these results together, we can determine that the G2019S mutation affects the immune compartment during infection, leading to dysregulated gene expression in neutrophils and monocytes.

To assess how the *Lrrk2* G2019S mutation may alter cell-cell communication during infection, we performed CellChat analysis comparing WT and *Lrrk2* G2019S mice. The overall number and strength of interactions was similar between WT and *Lrrk2* G2019S mice (Supplementary Fig. 6A, B). Further pathway-level analysis using rankNet revealed that *Lrrk2* G2019S mice displayed enhanced signaling strength across several pathways compared to WT (Supplementary Fig. 6C).

### *Lrrk2* G2019S mutation leads to increased colon histopathological damage after *C. rodentium* infection

**Fig. 4:**
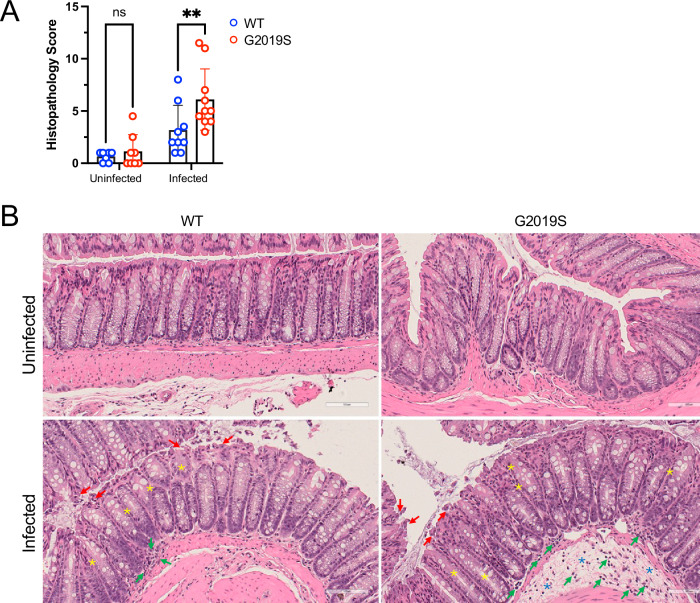
*Lrrk2* G2019S mutation leads to increased colon morphological damage after *C. rodentium* infection. Male and female *Lrrk2* G2019S and WT mice (male-to-female ratio = 0.43) were infected once with ~1 × 10^9^ CFU of *C. rodentium*. Colons were harvested 7 days after infection, fixed in 10% formalin for 24 h, washed in 70% ethanol, and diaphanized in xylene before embedding in paraffin. Sections were stained with Hematoxylin and Eosin (H&E) and scored for histopathology. **A** Total histopathology score. **B** H&E representative images. Data are presented as mean ± SD and analyzed by two-way ANOVA with Fisher’s LSD post-test. **p < 0.01. *n* = 7–11 mice per group. Two independent experiments were done and showed as a pool. White bars represent 100 µm. Symbols: blue star, edema; green arrow, inflammatory infiltrate; red arrow, erosion; yellow star, goblet cell loss. Please, also see Supplementary Fig. 8.

Given the differences in innate immunity, we assessed tissue-level effects of the G2019S mutation, post-infection. We performed histopathological analysis of colonic tissue from infected WT and G2019S mice on day 7 p.i. Uninfected control colons showed minimal histopathological scores with no differences between genotypes. Colons from infected mice demonstrated elevated inflammatory infiltrate, erosion, goblet cell loss, and edema—features more pronounced in G2019S mice, which had significantly higher total histopathological scores. (Fig. 4A, B and Supplementary Fig. 7A–H). Together, these results suggest that *Lrrk2* G2019S mice undergo a more severe inflammatory process in the colon tissue compared to WT mice during infection.

### *Lrrk2* G2019S mutation leads to a cell-intrinsic increase in neutrophil effector function that is kinase-dependent

**Fig. 5:**
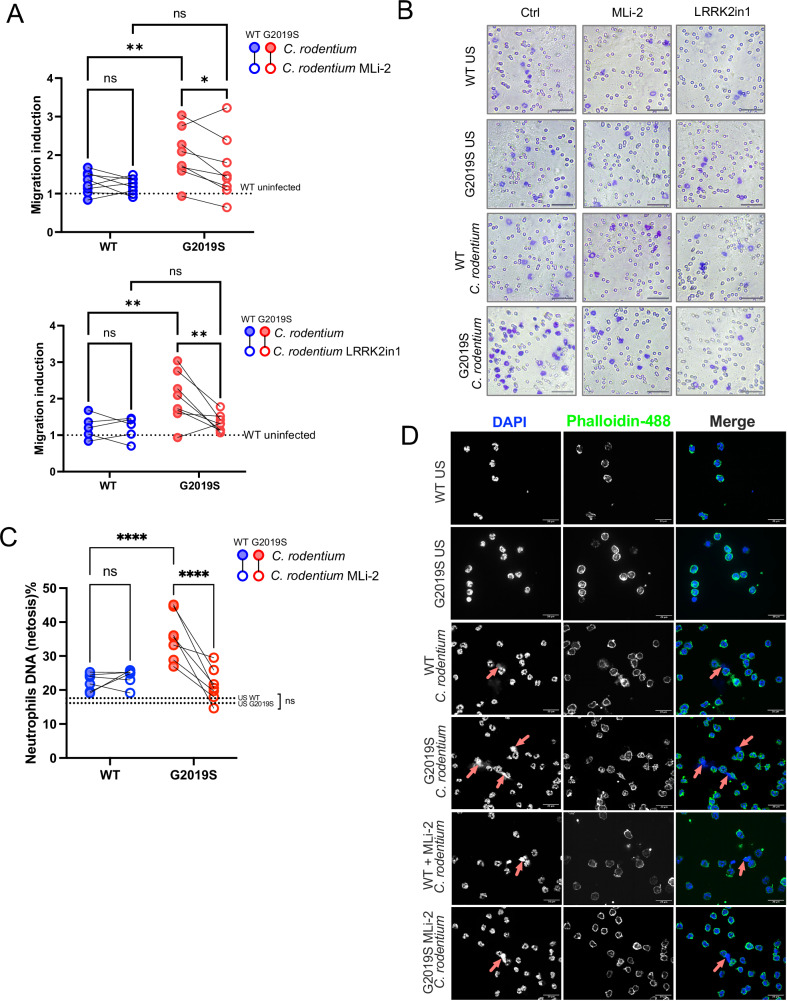
*Lrrk2* G2019S mutation leads to a cell-intrinsic increase in neutrophil effector function that is kinase-dependent. Whole blood, femurs, tibias, and humeri were collected from male and female *Lrrk2* G2019S and WT mice (male-to-female ratio = 0.55). **A**, **B** Bone marrow neutrophil migration quantification and representative images. Ly6G-isolated neutrophils were isolated from the bone marrow and incubated for 1 h with MLi-2 (100 nM), LRRK2in1 (1 μM), or DMSO (vehicle control) at 37 °C. Cells were seeded at 2 × 10^5^ cells per 5 μm transwell and allowed to migrate towards *C. rodentium* (MOI = 100) or PBS control for 1 h at 37 °C with 5% CO_2_. Four random fields of view were obtained per membrane, quantified by a blind experimenter, and averaged per mouse. Data are presented as mean ± SD and analyzed by two-way ANOVA with Fisher’s LSD post-test. *p < 0.05, **p < 0.01, *n* = 5–7 mice per group. A pool of two independent experiments is presented, normalized to WT untreated, uninfected controls for each respective experiment. Scale bar = 50 μm. **C**, **D** Blood neutrophil NETosis. Ly6G-isolated neutrophils were added at 1 × 10^5^ to a 24-well plate with coverslips. Cells were treated with MLi-2 (100 nM) or DMSO (vehicle control) for 1 h at 37 °C with 5% CO_2._ Neutrophils were stimulated with *C. rodentium* and incubated for 2 h at 37 °C with 5% CO_2_. Cells were stained for DAPI (blue) and Phalloidin (Alexa Fluor 488). Nine random fields of view from each condition were gathered, quantified results are shown as the percentage of NETosed cells. Data are presented as mean ± SD and analyzed by two-way ANOVA with Fisher’s LSD post-test. ****p < 0.0001. *n* = 6–7 mice per group. A pool of two independent experiments is presented. Scale bar = 20 μm.

Given the increased presence and dysregulated gene expression of neutrophils in infected *Lrrk2* G2019S mice, together with previous data indicating neutrophils have high LRRK2 protein expression and activity^33–35^, we wanted to determine if the G2019S mutation drove cell-intrinsic differences in neutrophils and their responses to infectious or inflammatory stimuli. Notably, several of the DEGs uncovered in the scRNAseq data have previously been implicated in neutrophil effector functions such as migration^32^ and NETosis^36^. A 1-h migration assay using purified bone marrow neutrophils (Supplementary Fig. 8A) showed significantly more G2019S neutrophils migrating towards *C. rodentium* compared to WT neutrophils. This effect was reversed when treated with LRRK2 kinase inhibitors—MLi-2 or LRRK2in1^37–39^, indicating the effect is kinase dependent (Fig. 5A, B). Effective kinase inhibition was further validated through immunoblotting of phospho-LRRK2 and phospho-Rab 10 (a LRRK2 substrate) (Supplementary Fig. 9). NETosis was also significantly increased in G2019S peripheral blood neutrophils compared to WT, following exposure to *C. rodentium* (Fig. 5C, D, Supplementary Fig. 8B), and reversible with MLi-2 treatment. Taken together, these results indicate that the *Lrrk2* G2019S mutation has a profound cell-intrinsic impact on neutrophil function, resulting in an overall increase in neutrophil effector mechanisms that are kinase-dependent.

### Colonic infection in *Lrrk2* G2019S mice initiates a skewing towards Th17 CD4^+^ T cells

**Fig. 6:**
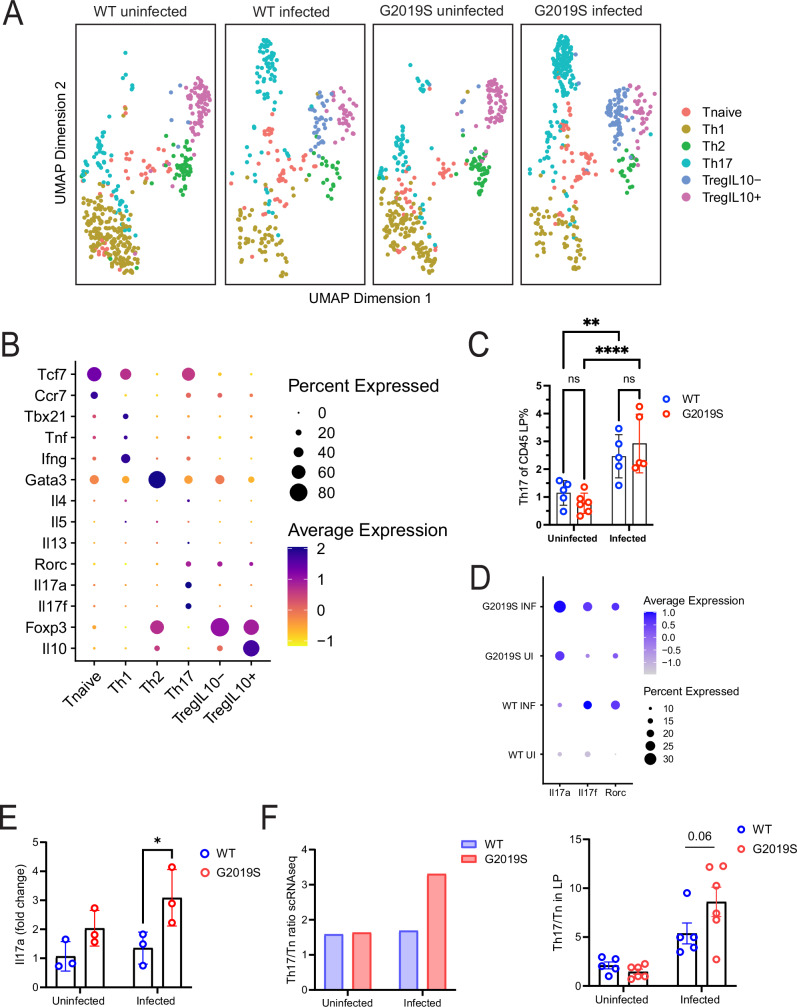
Colonic infection in *Lrrk2* G2019S mice initiates a skewing towards Th17 CD4^+^ T cells. Male and female *Lrrk2* G2019S and WT mice were gavaged once with ~1 × 10^9^ CFUs of *C. rodentium*, and colons were harvested. Immune cells were isolated for scRNAseq. **A** Re-clustering of CD4 T cell population based on effector T cell identities, depicted in a UMAP plot. **B** CD4 T cell subset markers used to identify effector cells based on literature. scRNAseq was obtained from pooled conditions of *n* = 3 mice per group (male-to-female ratio = 0.5). One independent experiment is presented. **C** Percent Th17 (RORgt+) CD4 T cells of CD45+ cells isolated from the colonic lamina propria across conditions. Data are presented as mean ± SD and analyzed by two-way ANOVA with Fisher’s LSD post-test. **p < 0.01, ****p < 0.0001, *n* = 5–6 mice per group. One representative of two independent experiment is presented (male-to-female ratio = 0.9). **D** Dotplot depicting average expression of *Il17a*, *Il17f*, and *Rorc* transcripts within Th17 effector subset. **E** *Il17a* expression fold change of sorted CD45+ immune cells from the lamina propria measured through quantitative PCR (qPCR). One representative of two independent experiment is presented. *n* = 3 mice per group (male-to-female ratio = 0.5). Data are presented as mean ± SD and analyzed by two-way ANOVA with Fisher’s LSD post-test. *p < 0.05. All qRT-PCR data were normalized to *Gapdh* using the ΔΔCt method. **F** Left: Ratio of absolute counts of Th17 effector cells over T naïve cells in scRNAseq dataset. Right: Ratio of Th17 effector cells over T naïve cells in CD45+ lamina propria populations in flow cytometry data.

Recent studies have shown that neutrophils can regulate Th17 cells through the formation of NETs, which can cause tissue damage and prolonged inflammatory response^40,41^. Th17 cells also play a key role in response to *C. rodentium* infection^42–44^. We further investigated CD4+ T cell subsets in our scRNAseq dataset. Using unsupervised clustering, we identified six distinct subsets—T naïve, Th1, Th2, Th17, and IL-10-negative and positive T regulatory cells (Treg IL10^−^ and Treg IL10^+^) in accordance with established transcriptional markers for CD4+ T cells^45^ (Fig. 6A, B).

At baseline, no genotype differences between cell clusters were identified. However, following infection, Th17 cells demonstrated a trend towards increase in *Lrrk2* G2019S-infected mice compared to WT infected (Fig. 6A). This trend was further supported using flow cytometry (Fig. 6C). To identify if there is a greater Th17 effector skew in *Lrrk2* G2019S-infected mice, we looked at the average expression of *Il-17a* and *Il-17f*, two major cytokines produced by Th17 cells^46^. Additionally, we assessed transcriptional factor RAR-related orphan receptor gamma (RORyt), encoded by *Rorc*, which is a critical regulator of Th17 differentiation, inducing expression of *Il-17a* and *Il-17f*^40,47^. We saw tendencies for increased *Il17a* expression in the Th17 cluster of *Lrrk2* G2019S-infected mice, compared to other conditions (Fig. 6D). Reverse transcriptase quantitative PCR (RTqPCR) of CD45^+^ immunocytes from the colonic lamina propria further demonstrated a significant upregulation of *Il17a* in G2019S-infected mice (Fig. 6E). To better understand if a skew towards Th17 is occurring, we also looked at the Th17/T naive ratio. Through both scRNAseq and flow cytometry, we saw a tendency to increase in this ratio in *Lrrk2* G2019S-infected mice (Fig. 6F).

## Discussion

LRRK2 expression and activity are enriched in immune cells, and *LRRK2* mutations are common risk factors for both PD and IBD, placing LRRK2 at the intersection of the immune system, the intestine, and PD^10,21,48–50^. In the present study, we have systematically investigated the early immune response to intestinal infection in the context of PD-associated LRRK2 G2019S. At day 7 post-*C. rodentium* infection, we observed increased accumulation of neutrophils in G2019S mouse colon, compared to WT colon. This coincided with elevated tissue pathology and inflammation driven by increased erosion, edema, and goblet cell loss scores. Functionally, G2019S neutrophils showed cell-intrinsic increases in chemotaxis and NETosis under inflammatory conditions compared to WT controls, in a kinase-dependent manner. Infected G2019S mice also demonstrated a skewing of CD4 subsets towards a Th17 profile, with increased transcriptional levels of *Il-17a*. Together, these results point to a role for LRRK2 in immune cells during the earliest responses following intestinal infection. Although *Lrrk2* mRNA expression was low in neutrophils, transcript levels may not accurately reflect protein abundance or activity in this cell type. Neutrophils in the small intestine have been shown to exhibit the highest LRRK2 protein activity, as measured by Rab10 phosphorylation, compared to other cell types^35^, and show high protein levels and activity in the peripheral blood^34^.

Prior work has emphasized the effects of G2019S on monocytes, macrophages, and microglia^51^. Fewer studies have examined the effects of G2019S in the context of neutrophils. Here, we demonstrated that during gram-negative intestinal infection, G2019S neutrophils have increased presence in the lamina propria and display a distinct transcriptional profile characterized by heightened type I and II IFN responses compared to WT mice. This is consistent with previous reports that the G2019S mutation is associated with increased IFN signaling under inflammatory conditions^51^. Moreover, *LRRK2* expression is consistently induced by IFN-γ stimulation^17,18,52,53^, further supported by the presence of IFN response elements in the *LRRK2* promoter region^17^. Overall, demonstrating a close link between LRRK2 and IFN pathways, both relevant in the context of PD and immunity.

LRRK2 kinase activity and G2019S-mediated effects on migratory function have previously been characterized in microglia, macrophages, and neutrophils, with conflicting results^53–55^. Moehle et al. demonstrated that G2019S myeloid cells have enhanced chemotaxis in in vitro and in vivo migration assays towards thioglycolate^54^, and Panagiotakopoulou et al. similarly observed increased migration in G2019S iPSC-derived microglia towards a chemoattractant, adenosine triphosphate^53^. Results from Mazaki et al. in dH-L60-cell line-derived neutrophils indicated *LRRK2* germ-line silencing and siRNA knockdown both decreased neutrophil migration towards fMLP. However, inhibition of LRRK2 kinase activity with MLi-2 increased the migratory capacity of WT cells^55^ indicating that acute suppression of LRRK2 activity can increase migration in some scenarios. Our results show that two LRRK2 inhibitors, MLi-2 and LRRK2in1, decreased the elevated migration of G2019S neutrophils after infection, while having no significant effects on WT neutrophils. While the dH-L60 cell line might behave differently than ex vivo WT mouse neutrophils, we found LRRK2 inhibition effects were clear in infected G2019S mouse neutrophils.

Neutrophils and Th17 cells communicate bidirectionally, but the specific dynamics of their interaction mediated by G2019S during infection remains unclear. Th17 cells are potent recruiters of neutrophils to sites of inflammation indirectly via IL-17A. Neutrophils can regulate recruitment of Th17 cells through release of CCL20, CCL2, and CXCL10 in response to IFNγ and LPS^56^. NETs can also drive Th17 polarization in a dose-dependent manner^57^, whereby histones decorating released NETs activate toll-like receptor 2 in naïve T cells, phosphorylate STAT3, and facilitate Th17 differentiation^57^. In *C. rodentium* infection kinetics, neutrophils are one of the first immune cells recruited to the colonic lamina propria, followed by T cells, including Th17 cells^24^. Therefore, we hypothesize that in G2019S mice, increased neutrophil presence and NETosis may promote Th17 differentiation and play into a positive feedback loop with neutrophil recruitment. To clarify the sequence of events, further analyses are needed. Park et al. previously showed that BAC humanized *LRRK2* G2019S overexpressing rats had an increased presence of immature myeloid cells with suppressive activity on Th17 cell differentiation in the gut, in response to acute (trinitrobenzene sulfonic acid (TNBS)-induced) and chronic (DSS-induced) colitis, relative to background strain rats^58^. Here, we use *C. rodentium* infection, which induces a more robust Th17 response than DSS-induced colitis. We demonstrated that the Th17 skewing was more pronounced in *Lrrk2* G2019S knock-in mice than their WT littermate controls, suggesting LRRK2-mediated Th17 skewing may be dependent on the inflammatory setting.

Understanding gene-environment interactions is critical to uncover the complex etiology of PD. Wallings et al. demonstrated that *Lrrk2* R1441C knock-in mice (a similar PD LRRK2 kinase mutation) exhibit age-dependent dysfunction in peritoneal macrophages. Young R1441C knock-in female mice showed increased IFNγ-dependent antigen presentation, cytokine release, and phagocytosis, while aged mice of both sexes developed immune cell exhaustion, marked by reduced antigen presentation and hyperphagocytosis^59^. Our previous work showed that repeated intestinal infection with *C. rodentium* in mice harboring a mutation in PD-related gene (*Pink1*^*−/−*^) triggered the development of PD-relevant dopamine-sensitive motor symptoms later in life. The study also revealed the infection-initiated presentation of mitochondrial antigens by antigen-presenting cells, which triggered the development of cytotoxic CD8+ T lymphocytes specific to mitochondria in both the brain and the periphery^60^. In follow-up studies, we mapped the early immunological events during *C. rodentium* infection in the colons of *Pink1*^−/−^ mice. At the peak of the innate immune response to *C. rodentium* (1-week p.i.), *Pink1*^−/−^ mice had dysregulated differentially expressed genes within myeloid cells of the monocyte/macrophage lineage^30^. Specifically, during infection, *Pink1*^−/−^ monocytes skewed towards “mature” macrophage- and DC-like profiles, with an increased capacity of MHC II presentation^30^. Like *Pink1*^−/−^, we also demonstrate that the monocytes in G2019S mice were affected 1-week post-*C. rodentium* infection. However, our results in G2019S mice showed increased immature-Ly6C^High^ monocytes in the colonic lamina propria, with downregulation of MHC II expression, distinct from the differences seen in the *Pink1*^*−/−*^ mice. Although the mechanisms regulating monocytes differ between *Lrrk2* G2019S (a common genetic variant linked to increased risk of PD) and loss-of-function mutations in PINK1 (rare recessive mutations associated with early-onset PD)^61^, our collective work highlights the involvement of two distinct PD-related genes in reprogramming of the immune response to infection.

While we are characterizing the earliest colonic responses to infectious colitis, others have explored G2019S in DSS models^22,62,63^. In agreement with our findings, Cabezudo et al. demonstrate that G2019S mice under DSS treatment have significantly elevated colonic histopathological score compared to WT DSS, with intense infiltration of leukocyte populations, and increased presence of MPO+ cells. Using WT bone marrow transplantation into G2019S mice, they could fully rescue the inflammatory phenotype, while inhibition of LRRK2 G2019S kinase activation partially rescued the phenotype. This indicates the clear involvement of LRRK2 kinase activity in immune cells and exacerbation of inflammatory phenotypes in G2019S mice during DSS-induced colitis. Others have also demonstrated that *Lrrk2* G2019S can render mice more vulnerable to DSS-induced colitis, or downstream consequences^22,63^. Our work extends these findings through assessment of the early response to a self-limiting infection with a natural mouse intestinal pathogen. This work reveals that endogenous LRRK2 G2019S impacts the very early response to infection in the gut, and that amongst all cells of the lamina propria, neutrophils are arguably the most prominently affected by the mutation in this setting. Furthermore, we uncover a number of cell-intrinsic differences in *Lrrk2* G2019S neutrophils, suggesting that these cells may play a primary role in the overall immune dysfunction observed in infected *Lrrk2* mutant mice.

Others have demonstrated that chronic treatment of G2019S mice with DSS led to development of PD-like motor impairments^22,63^ and that DSS coupled with injection of a human alpha-synuclein overexpression vector led to PD-like neurodegeneration in G2019S mice^62^. We did not observe any development of motor symptoms in the G2019S or WT mice within the time frame of our experiments. This is not surprising since both in our previous studies in *Pink*^*−/−*^ mice and in G2019S DSS colitis studies, motor symptom development required months of time and multiple exposures to infectious or inflammatory stimuli. While beyond the scope of this paper, it would be of interest to determine if repeated *C. rodentium* exposure and/or aging of the mice would lead to development of PD-like motor symptoms in *Lrrk2* G2019S knock-in mice. Recent work in a neurotoxic PD model showed that *C. rodentium* infection exacerbates motor symptoms when coupled with injection of the neurotoxin MPTP, compared to infection or MPTP alone. *C. rodentium* infection also caused decreased colonic dopamine and serotonin levels between days 7–14 p.i^64^., providing additional mechanistic gut-brain axis links.

While we are contributing to a growing body of literature uncovering the role of LRRK2 G2019S in immune regulation, our study is subject to certain limitations. Our findings provide insights in how *Lrrk2* G2019S may influence neutrophil function, but the precise mechanisms remain to be determined. Prior work suggests that increased migration in LRRK2 G2019S in myeloid cells may be regulated through binding of actin regulatory proteins responsible for controlling chemotaxis, whose interaction is blocked by LRRK2 kinase inhibitors^54^. Notably, one of the most significantly upregulated DEGs in our neutrophil dataset is *Actg1*, which encodes gamma-actin and critical for cell movement^65^. Further studies are required to validate actin involvement in our observed migration phenotype. A second limitation is that we did not establish the mechanistic link between increased neutrophil presence and NETosis on Th17 polarization in G2019S-infected mice. A neutrophil depletion assay could help clarify this sequence of events. With respect to our scRNAseq dataset, the analysis was only completed on one cohort of mice. While our data revealed clear and biologically relevant transcriptional changes, future studies are necessary to continue validating and extending our findings. Similarly, the effect sizes we can detect in many of our experiments are limited by the number of animals and tissues we can process in a timely manner, as well as the number of cells we can isolate from each sample. As a result, there may be more subtle effects of the *Lrrk2* G2019S mutation that remain undetected due to constraints in statistical power. Finally, an intrinsic limitation of using the *Lrrk2* G2019S knock-in mouse model^66,67^ lies in species-specific differences between human and mouse LRRK2 promoter regions and protein activity. Human LRRK2 exhibits greater kinase activity at steady state compared to mouse LRRK2^68^. Nonetheless, conserved IFN-γ response elements in the *LRRK2* promoter across species suggest this regulatory mechanism may be preserved^17^.

Taken together, our results demonstrate that the *Lrrk2* G2019S mutation has a profound impact on the early response to gut infection. This is evident by the increase of neutrophil migration, increase in NETosis, inflammatory-mediated tissue damage, and elevation of *Il-17a* expression. Considering that immune dysregulation plays a role in the development and progression of neurodegenerative disease, our findings could contribute to a better understanding of mechanisms within the prodromal phase of PD. This understanding could inform the development of pharmacological targets for early detection and intervention in PD.

## Methods

Protocols associated with this work can be found on protocols.io: 10.17504/protocols.io.bp2l6y5ervqe/v2.

### Ethics statement and mice

All animal experiments were performed in compliance with the guidelines and conditions specified by the Canadian Council on Animal Care and were approved by the animal care committee of McGill University (Animal Use Protocol number MCGL-5009). Twelve- to sixteen-week-old male and female *Lrrk2* homozygous knock-in G2019S (RRID:IMSR_JAX:030961)^66,67^ and their respective wild-type (WT) littermate mice were used. Mice were bred as heterozygotes and were maintained under specific pathogen-free conditions at the animal facility of McGill University. Sample sizes were based on previous similarly designed experiments from our research group. Exact mouse numbers for each experiment are included in the figure legends. Mice were randomly assigned to different experiments. Mice sharing the same cage received the same type of treatment—either Luria-Bertani broth (LB) control or *C. rodentium* infection. Animal studies were not blinded. Histopathology scoring, multiplex cytokines and chemokines, and neutrophil migration and NETosis counting were conducted blindly.

### *Citrobacter rodentium* infection

The chloramphenicol-resistant *C. rodentium* strain DBS100^69^ was used in this study for all in vivo and in vitro experiments. *C. rodentium* inocula were prepared by culturing bacteria overnight with shaking at 37 °C, 5% CO2 in LB. Bacteria were washed and resuspended in LB, and the bacterial density was determined by optical density (O.D.) measured at 600 nm with a spectrophotometer. Mice were inoculated by oral gavage with ~10^9^ CFU of *C. rodentium* diluted in 100 μL of LB. Control mice received 100 μL of LB. Inoculum was confirmed by serial dilutions and plating. The body weight of mice was monitored, and feces were collected at different time points after infection to measure pathogen shedding.

### Quantification of *C. rodentium* burden

On days 4, 7, 8, 12, 21, and 28 p.i., fresh fecal samples were collected, diluted in 1 mL PBS, and homogenized by bead-beating with 1 mm ceramic beads once for 40 s at 6000 rpm using a MagNA Lyser (Roche Diagnostics GmbH). After 7 days of *C. rodentium* infection, mice were euthanized, and colons were harvested. Their lengths were measured and cut perpendicularly into five segments for various assays (from distal to proximal): histology, CFU, mRNA, cytokine, and MPO assay. Colonic samples for CFU calculation were then homogenized mechanically (Polytron PT 2100) in PBS. To determine CFUs, serial dilutions of homogenized samples were plated on MacConkey agar plates with 100 μg/mL chloramphenicol. Plates were incubated at 37 °C overnight before counting. *C. rodentium* was identified by its unique colony morphology, and CFUs were calculated after normalization to the weight of each fecal or tissue sample.

### Constipation and gut motility measurement

Gastrointestinal transit was assessed by measuring fecal water content and the number of fecal pellets expelled. Briefly, on day 5 or 7 p.i., G2019S and WT mice were placed individually in cages without bedding or food. To measure fecal water content, the first two fecal pellets were collected in a pre-weighed 1.5 mL tube. The tube was weighed immediately after collection, then left to dry overnight at room temperature in a biosafety hood. The final weight was recorded the following day, and water content was calculated as the difference between wet and dry weights, divided by the wet weight. To assess fecal pellet output, the number of fecal pellets produced by each animal in a 2-h interval was recorded.

### Histopathological analysis

Colon samples were collected after euthanasia and fixed in 10% buffered formalin. Blocks were embedded in paraffin and sectioned at 4-μm using a microtome. Tissue sections were deparaffinized with xylene, rehydrated in ethanol, and stained with hematoxylin and eosin (H&E) at the histology innovation platform of the Rosalind and Morris Goodman Cancer Institute of McGill University. Slides were scanned at 20× magnification, and images were captured using a Leica Aperio slide scanner (Leica). Histopathological scoring was performed blindly by an expert veterinary pathologist based on the scoring criteria of colon lesions^70^. They were scored based on inflammatory infiltrate (0–4), polymorphonuclear (PMN) cell infiltrate (0–4), loss of crypts (0–2), proportional loss of goblet cells (0–2), edema (0–1), erosion or ulceration (0–3), hemorrhage (0–2), and necrosis (0–1).

### Immunofluorescence and confocal microscopy

Immunofluorescent staining and confocal microscopy were performed on paraffin sections. The slides were deparaffinized with xylene three times for 3 min. The sections were rehydrated in 100% ethanol three times for 3 min, followed by one time with 95% ethanol and washed in deionized (DI) water. The slides were then incubated for 20 min in boiling 0.1 M citrate buffer (pH 6.0) for antigen retrieval, followed by a 20 min incubation in the same solution at room temperature (RT). Then, samples were rinsed in DI water. For staining, slides were blocked with a blocking buffer of 3% bovine serum albumin (BSA)(Sigma, Cat# A2153-100G) and Fc-Block (anti-mouse CD16/CD32) 1:100 (Invitrogen, Cat# 14-01061-86) in PBS for 3 h at room temperature. Tissues were incubated with the antibody Ly6G-AF594, clone: 1A8 (BioLegend, Cat# 127636) at 1:200 dilution in 1% BSA-PBS for 16–18 h at 4 °C. The next day, samples were washed 3 times with PBS and stained with DAPI (Invitrogen, Cat# D1306) 1:5000 for 10 min at room temperature, followed by 3 more washes with PBS. Tissues were mounted using a Prolong Diamond antifade (Invitrogen, Cat# P36961). Sections were viewed on a Zeiss AXIO confocal microscope.

### Myeloperoxidase (MPO) assay

Neutrophil accumulation in the colon was assessed by MPO activity. Proximal colon samples were harvested after euthanasia, homogenized in a buffer (0.1 M NaCl, 0.02 M NaPO₄, 0.015 M NaEDTA, pH 4.7) (Polytron PT 2100), and centrifuged at 260 × *g* for 10 min. Pellets underwent hypotonic lysis with 0.2% NaCl solution, followed by addition of 1.6% NaCl and 5% glucose solution. Samples were centrifuged again and resuspended in 0.05 M NaPO₄ (pH 5.4) containing 0.5% hexadecyltrimethylammonium bromide and re-homogenized. Samples were then subjected to three freeze–thaw cycles in liquid nitrogen, centrifuged, and the supernatants were collected. MPO enzymatic activity was measured by changes in OD 450 nm using tetramethylbenzidine (1.6 mM) and H₂O₂ (0.5 mM). Results were expressed as ΔOD per gram of colon tissue.

### Colonic lamina propria cell preparation

Immune cells from the colonic lamina propria were isolated as previously described^30^. Briefly, following euthanasia, whole colons were harvested and placed in 50 mL tubes containing 20 mL of ice-cold PBS (Wisent, Cat# 311-010-CL). After flushing out fecal material and removing fat, the colons were cut longitudinally, then perpendicularly into 2-cm sections. To separate epithelial cells from the lamina propria, tissue sections were incubated in 2 mM EDTA-PBS for 15 min at 37 °C (Invitrogen, Cat# 15575-038), followed by three rounds of vigorous manual shaking until the cellular suspension appeared cloudy. The remaining tissue sections, consisting of the lamina propria, were digested twice enzymatically using a 2:1 ratio of collagenase from *Clostridium histolyticum* (MilliporeSigma, Cat# C5138-1G) and DNase I (MilliporeSigma, Cat# 10104159001). Each digestion was incubated for 30 min at 37 °C with vigorous vortexing every 15 min. Following each enzymatic digestion, the colon tissues were strained through a 70-μm cell strainer to isolate the immune cells, then spun down at 450 × *g* at 4 °C for 5 min. The pellet was then resuspended in Fluorescence-Activated Cell Sorting (FACS) buffer (2% FBS-PBS, 1 mM EDTA).

### Flow cytometry

Following isolation, cells were stained with 1:2000 of Zombie red fixable viability dye (Biolegend, Cat# 423109) in PBS for 20 min on ice. Next, Fc-Block (anti-mouse CD16/CD32) 1:100 (Invitrogen, Cat# 14-01061-86) was added, and surface markers were stained with a mixture of conjugated antibodies 1:250 in FACS buffer (2% FBS-PBS, 1 mM EDTA) with 10% Brilliant Stain Buffer Plus (BD, Cat# 566385) for 30 min on ice. For transcriptional factor (TF) staining, cells were fixed in Foxp3/Transcription Factor Staining Buffer (eBiosciences, Cat# 00-5523-00) for 45 min on ice, then washed with permeabilization buffer and stained with a mixture of conjugated antibodies diluted 1:125 in the permeabilization buffer (listed in Table 3). Single-stained cells of the appropriate processed tissue were used as a reference control for unmixing. Fluorescence minus one controls (FMOCs) were used to set gating for positive populations. TF UltraComp eBeads (Invitrogen, Cat# 01-2222-42) were used as a single stain control of transcriptional factors and lowly expressed markers. Autofluorescence was deducted using unstained lamina propria cells. Samples and their respective controls were acquired using the spectral cytometer Aurora 4L (Cytek). Flow Cytometry Standard (FCS) files were exported and analyzed using FlowJo Software v10.9 (BD, RRID:SCR_008520, https://www.flowjo.com/solutions/flowjo).

**Table 3:** List of antibodies used in flow cytometry

Target	Fluorochrome	Manufacturer	Clone	RRID
CD45	Brilliant Violet 785	Biolegend	30-F11 (C) & (F)	AB_2564590
TCRb	Brilliant Violet 510	Biolegend	H57-597 (A)	AB_2562349
CD4	Brilliant Violet 570	Biolegend	RM4-5 (F)	AB_10897943
CD8a	Spark Blue 550	Biolegend	53-6.7 (F)	AB_2832268
CD44	Brilliant Violet 605	Biolegend	IM7 (B)	AB_2562451
CD103	PerCP e-fluor 710	ThermoFisher	2E7 (B)	AB_2573704
CD69	PE	Biolegend	H1.2F3 (A)	AB_313110
Tbet	Brilliant Violet 711	Biolegend	4B10 (H)	AB_11218985
Gata3	E-fluor 660	ThermoFisher	TWAJ (H)	AB_10596663
FoxP3	PerCP-Cy5.5	ThermoFisher	FJK-16s (H)	AB_914351
Rorγt	PE-efluor 610	ThermoFisher	Q31-378 (H)	AB_2574650
CD19	PE-fire 640	Biolegend	6D5 (F)	AB_2888725
MHC-II	APC-efluor 780	ThermoFisher	M5/114.15.2 (A)	AB_1548783
CD11c	PE-fire 810	Biolegend	QA18A72 (F)	AB_2904307
CD11b	APC-fire 810	Biolegend	M1/70 (F)	AB_2910274
PDL1	Brilliant Violet 421	Biolegend	10F.9G2 (A)	AB_10897097
CD86	Alexa fluor 700	Biolegend	GL-1 (A)	AB_493720
Ly6C	APC	Biolegend	HK1.4 (B)	AB_1732076
Ly6G	Spark Yellow-Green 593	Biolegend	1A8 (F)	AB_2892282
Ly6G	FITC	Biolegend	1A8 (F)	AB_1236488
Ki67	PE-Cy7	Biolegend	11F6 (H)	AB_2910305
EpCam	E-fluor 450	ThermoFisher	390 (B)	AB_10717090
CX3CR1	Brilliant Violet 650	Biolegend	SA011F11 (E)	AB_2565999
Nk1.1	PE-Fire 700	Biolegend	S17016D (F)	AB_2910320
CD107a	BV510	Biolegend	1D4B	AB_2783064

### Single-cell RNA sequencing

scRNAseq was completed using 10x Genomics Chromium sequencing. Colonic lamina propria cells were isolated and pooled from three mice per condition (WT uninfected, WT infected, G2019S uninfected, G2019S infected). Using the Single Cell 3′ Reagent kit (V3.1 assay, https://www.10xgenomics.com/support/single-cell-geneexpression), 40,000 cells were loaded on the Chromium per the manufacturer’s instructions. Reverse transcription (RT), cDNA synthesis and amplification, and library preparation were completed as previously described^30^. Briefly, cells were first partitioned on a nanoliter-scale using barcoded Gel Bead-In-Emulsions (GEMs). After the GEM generation, Gel 39 Beads were dissolved, primers were released, and co-partitioned cells were lysed. Then, cellular transcripts were reverse transcribed with primers containing (1) TruSeq sequence, (2) 16 nt 10x Barcode, (3) a 12 nt unique molecular identifier (UMI), and (4) a 30 nt poly(dT) sequence, which became mixed with the cell lysate and Master Mix containing RT reagents. The incubation resulted in barcoded, full-length cDNA from poly-adenylated mRNA. After that, Silane magnetic beads were used to purify the cDNA from the RT reaction mixture, followed by the amplification of cDNA through PCR, creating a library. Sequencing was performed using NovaSeq 6000 S4 PE 100 bp, resulting in a final output of on average, 20,000 reads/cell, which were then processed using 10X Genomics Cell Ranger Single Cell 2.0.0 pipeline (RRID:SCR_017344, https://support.10xgenomics.com/single-cell-gene-expression/software/pipelines/latest/what-is-cell-ranger). FASTQs outputs were aligned to the mouse GRCm38.p5 reference genome. Each sample had been assigned Gene-Barcode matrices by counting UMIs and filtering non-cell-associated barcodes. Finally, Seurat V5.1.0 (RRID:SCR_016341, https://satijalab.org/seurat/get_started.html) R toolkit V4.4.1 (RRID:SCR_001905, https://www.r-project.org/) was used for quality control and downstream analysis of the scRNAseq data. Each Seurat object was identified with default parameters (min. cells = 3, min. features = 200), and low-quality cells and doublets were excluded based on gene counts and percent of mitochondrial genes (downstream analyses were performed on cells with gene counts between 200 and 2500, and percent of mitochondrial genes fewer than 5)^71^. Gene expression was log-normalized to a scale factor of 10,000. Both Seurat objects (uninfected and infected) were then integrated as previously described in ref. ^28^.

### mRNA extraction and quantitative RT-PCR

RNA from isolated colonic immunocytes was extracted using a Pure Link RNA kit (Invitrogen, Cat# 12183025) according to the manufacturer’s instructions. RNA was reverse transcribed using SuperScript IV VILO RT (Invitrogen, Cat# 11756050) per the manufacturer’s instructions. Quantitative PCR was performed using Taqman Fast Advanced Master Mix (Applied Biosystems, Cat# 4444963). Probes are listed in Table 4. qPCR was performed on StepOnePlus (Applied Biosystems, USA). CT values were analyzed using the formula 2^−ΔΔCt^, normalizing target gene expression to *Gapdh*.

**Table 4:** List of probes used for qPCR

Target	Manufacturer	Cat #
Il17a	Taqman	Mm00439618_m1
Gapdh	Taqman	Mm99999915_g1

### Bone marrow and blood neutrophil isolation

Uninfected WT and G2019S mice were euthanized using CO_2_ affixation; femurs, tibias, and peripheral blood (cardiac puncture) were harvested. Neutrophils were isolated from femurs and tibias by flushing with PBS using a 27 G needle and syringe. Cells were pelleted through centrifugation at 450 × *g* for 5 min at 4 °C. Red blood cells (RBCs) were lysed using lysis buffer (Biolegend, Cat# 420301, diluted to 1X with sterile water) per the manufacturer’s instructions. Peripheral blood neutrophils were isolated through repeated treatments with lysis buffer (Biolegend, Cat# 420301) to clear RBCs. Cell counts for bone marrow and blood neutrophils were recorded using a hemacytometer. Cells were pelleted through centrifuging at 450 × *g* for 5 min at 4 °C. Cells were resuspended in 90 µL/10^7^ of isolation buffer (PBS containing 2% fetal bovine serum (FBS) and 2 mM EDTA) and 10 µL/10^7^ of mouse anti-Ly6G MicroBeads (Miltenyi Biotec, Cat# 130-120-337), then incubated at 4 °C for 10 min. Cells were washed with 1 mL of isolation buffer per 10^7^ cells and pelleted through centrifuging at 450 × *g* for 5 min at 4 °C. Cells were resuspended in 500 µL of isolation buffer and passed through magnetic-activated cell sorting (MACS) LS columns according to the manufacturer’s instructions (Miltenyi Biotec, Cat# 130-042-401).

### LRRK2 kinase inhibition

Post magnetic sorting, cells were counted, resuspended in Dulbecco’s Modified Eagle’s Medium (DMEM) with 10% FBS, and seeded in 24-well plates with 1 mL of media. Cells were treated for 1 h in an incubator at 37 °C and 5% CO_2_ with either 100 nM of MLi-2 (Cayman Chemical, Cat# 19305), 1 μM LRRK2-in-1 (Cayman Chemical, Cat# 18094), or DMSO as a vehicle control.

### Neutrophil purity assessment

The anti-Ly6G MicroBead isolation method used in this study typically yields unstimulated, viable neutrophils with a purity greater than 95%. To confirm neutrophil purity, isolated cells were stained with anti-mouse Ly6G conjugated to FITC **(**Biolegend, Cat# 127605) at a 1:250 dilution in 2% FBS-PBS with 1 mM EDTA and incubated on ice for 30 min. Samples were then washed with PBS and stained for viability with 7-AAD (Invitrogen, Cat# 00-6993-50). Samples and their respective controls were acquired using the FACS Diva software on the LSR Fortessa cytometer (BD). Unstained isolated neutrophils were used as an autofluorescence control. Flow Cytometry Standard (FCS) files were exported and analyzed using FlowJo Software v10.9 (BD).

### Immunoblotting

Cells were washed once with Tris-buffered saline (TBS) and lysed in Laemmli buffer. Protein concentration was determined using the EZQ™ Protein Quantitation Kit (Thermo Fisher, Cat# R33200). Equal amounts of protein were loaded onto 4%–15% pre-cast SDS-PAGE gels (Bio-Rad), separated by electrophoresis, and transferred to membranes using the Trans-Blot Turbo transfer system (Bio-Rad). Membranes were blocked for 30 min at room temperature in TBS containing either 5% non-fat dry milk or 5% bovine serum albumin (BSA), then incubated overnight at 4 °C with primary antibodies diluted in 5% BSA-TBS. Primary antibodies and concentrations are listed in Table 5. The next day, membranes were washed three times with TBS containing 0.1% Tween-20 (TBS-T), followed by a 1-h incubation at room temperature with HRP-conjugated secondary antibodies diluted in TBS-T supplemented with 5% milk or 5% BSA. After three additional washes in TBS-T, signal detection was performed using Clarity or Clarity Max Western ECL substrate (Bio-Rad), and chemiluminescence was visualized with the ChemiDoc imaging system (Bio-Rad).

**Table 5:** List of antibodies used for immunoblotting

Target	Manufacturer	Cat #	Concentration	RRID
LRRK2	Abcam	ab133474	1:1000	AB_2713963
p-LRRK2 S935	Abcam	ab133450	1:1000	AB_2732035
p-Rab10 T73	Abcam	ab241060	1:1000	AB_2884876
GAPDH	Millipore	MAB374	1:3000	AB_2107445

### In vitro neutrophil migration

2 × 10^5^ neutrophils isolated from the bone marrow, suspended in 150 µL of DMEM + 10% FBS, and pre-treated with either MLi-2, LRRK2-in-1, or DMSO vehicle control, were seeded in the upper chamber of transwell inserts with a 5 µm pore size (Corning, Cat# CLS3421). Sixty microliters of mouse serum from C57BL/6 mice were added to 540 µL of DMEM + 10% FBS in the lower chamber of the transwell. 1 uL of *C. rodentium* (OD600 = 1) was supplied to the lower chamber to obtain a multiplicity of infection (MOI) of 100, or 1 µL of PBS was added as a negative control. Neutrophils were given 1 h to migrate, incubated at 37 °C with 5% CO_2_. At the end of the incubation, transwells were moved to a clean 24-well plate, and the remaining liquid was aspirated. The transwell membranes were washed with 1× PBS once, residual non-migrated cells were gently removed using a cotton swab, and the membrane was fixed and stained using Kwik-Diff Stains (Epredia, Cat# 99-907-06) per the manufacturer’s instructions. Stained inserts were left to dry overnight. Membranes from dried inserts were removed by a scalpel, and the output side was mounted upright onto glass slides (Fisher, Cat# 22-037-246) using Permount Mounting Medium (Fisher Chemical, Cat# SP15-100). Membranes were imaged using a 10× objective on the Leica DSM1000 microscope. Five random fields of view were obtained per membrane and quantified by a blinded experimenter.

### In vitro NETosis assay

Sterile 12 mm circular coverslips (Fisher, Cat# 12-545-80) were placed in the wells of a 24-well tissue culture plate. 1 × 10^5^ neutrophils isolated from peripheral blood in 1 mL of 37 °C 10% FBS (Gibco) DMEM (Gibco) were added to the wells with coverslips. Following the LRRK2 kinase inhibition (see above methods) with 100 nM of MLi-2 (Cayman Chemical, Cat# 19305), 1.7 µL of *C. rodentium* (OD600 = 1) or PBS (negative control) was added to the wells. The treated cells were incubated for 2 h at 37 °C with 5% CO2. Coverslips were washed with PBS and fixed with 300 µL of 4% Paraformaldehyde (PFA, Electron Microscopy Sciences) for 30 min. The PFA was subsequently washed with PBS, and the coverslips were stained with DAPI (Invitrogen, Cat# D1306) at a dilution of 1:2500 and Phalloidin 488 (Invitrogen, Cat# 424201) at a dilution of 1:50 in 300 µL of PBS for 20 min. To prepare the slides, 20 µL of ProLong Gold antifade reagent (Invitrogen) was added to glass slides (Fisher), and the coverslips were mounted so that the adhered cells faced the glass slide. The glass slides were left to dry overnight. NETosis was evaluated based on DAPI-staining morphology, which was assessed for circularity versus irregularity. Neutrophils were imaged using the Nikon CSU-X1 spinning disk microscope by the Z-stack acquisition of nine fields of view from each sample. Following this, we performed semi-automated quantification of NETosis events using ImageJ V 2.14.0 (RRID:SCR_002285, http://fiji.sc) on Icy software^72^. Z-stacks were merged, images had their channels separated into DAPI and Alexa Fluor 488, and brightness and contrast were auto-calibrated. Then, we converted them into 8-bit format, and values for the minimal size and background were set. After that, files were made binary, and the number of total particles and their circularity and irregularity were manually counted for each sample. Circularity was indicated by a value of 1. NETosing cells were then validated manually, blinded with respect to the sample. Neutrophils were considered to have undergone NETosis when neutrophils formed either cloud-like structures or elongated filaments, which are characteristics of NETosis.

### Statistical analysis

Statistical analyses were performed using GraphPad Prism version 10.2.2 (RRID:SCR_002798, http://www.graphpad.com/). All data are presented as the mean ± SD and were analyzed using Two-way analysis of variance (ANOVA) followed by Šídák or Fisher’s LSD post-test to compare different groups. Student’s t test was used to compare two groups. *P* value < 0.05 was considered a significant difference and marked as *; *p* < 0.01 as **, *p* < 0.001 as ***, and *p* < 0.0001 as ****. Each experiment was done at least twice, with the exception of scRNAseq, with a minimum of three mice per group.

## Supplementary information

Supplementary Figures and Tables

## Data Availability

The data, protocols, and key lab materials that were used and generated in this study are listed in a Key Resource Table, including all pertaining identifiers, which will be deposited at Zenodo upon acceptance for publication [10.5281/zenodo.14291137]. The transcriptomics dataset has been deposited to [GEO Accession #: GSE283183; https://www.ncbi.nlm.nih.gov/geo/query/acc.cgi?acc=GSE283183]. Data cleaning, processing, analysis, and visualization were performed using GraphPad Prism and R. An earlier version of this manuscript was posted to bioRxiv on December 4, 2024, at [10.1101/2024.11.26.625468].

## Code availability

The underlying code generated for this study can be found in [https://github.com/gruenheid-lab/pei-2024-lrrk2_neutrophil].
