# Author Correction: *Lrrk2* G2019S mutation incites increased cell-intrinsic neutrophil effector functions and intestinal inflammation in a model of infectious colitis

**DOI:** 10.1038/s41531-026-01285-z

**Published:** 2026-03-13

**Authors:** Jessica Pei, Nathalia L. Oliveira, Sherilyn J. Recinto, Alexandra Kazanova, Celso M. Queiroz-Junior, Ziyi Li, Katalina Couto, Susan Westfall, Ahmed M. Fahmy, Camila Tiefensee-Ribeiro, Irah L. King, Austen J. Milnerwood, Michel Desjardins, Ajitha Thanabalasuriar, Jo Anne Stratton, Samantha Gruenheid

**Affiliations:** 1 Department of Microbiology and Immunology, McGill University, Montreal, QC Canada; 2 Aligning Science Across Parkinson’s (ASAP) Collaborative Research Network, Chevy Chase, MD USA; 3 Montreal Neurological Institute, McGill University, Montreal, QC Canada; 4 Departamento de Morfologia, Universidade Federal de Minas Gerais, Belo Horizonte, Brazil; 5 Department of Pharmacology and Therapeutics, McGill University, Montreal, QC Canada; 6 Département de pathologie et biologie cellulaire, Université de Montréal, Montreal, QC Canada

**Keywords:** Inflammation, Microbiology

Correction to: *npj Parkinson’s Disease* 10.1038/s41531-025-01077-x, published online 29 August 2025

In this article, the author had inadvertently proceeded with the scRNAseq data analysis with a data set where one normalization step was omitted. They have now redone the analysis with the inclusion of this normalization step. The original article has been updated.

