# Supplementary material for: Lrrk2 G2019S mutation incites increased cell-intrinsic neutrophil effector functions and intestinal inflammation in a model of infectious colitis
Source: NPJ Parkinsons Dis. 2025 Aug 29;11:267. doi: 10.1038/s41531-025-01077-x (PMC12397289; doi:10.1038/s41531-025-01077-x)
Supplement: Supplementary file 1 — Supplementary Figures and Tables [file 41531_2025_1077_MOESM1_ESM.pdf]

Supplemental Figures.

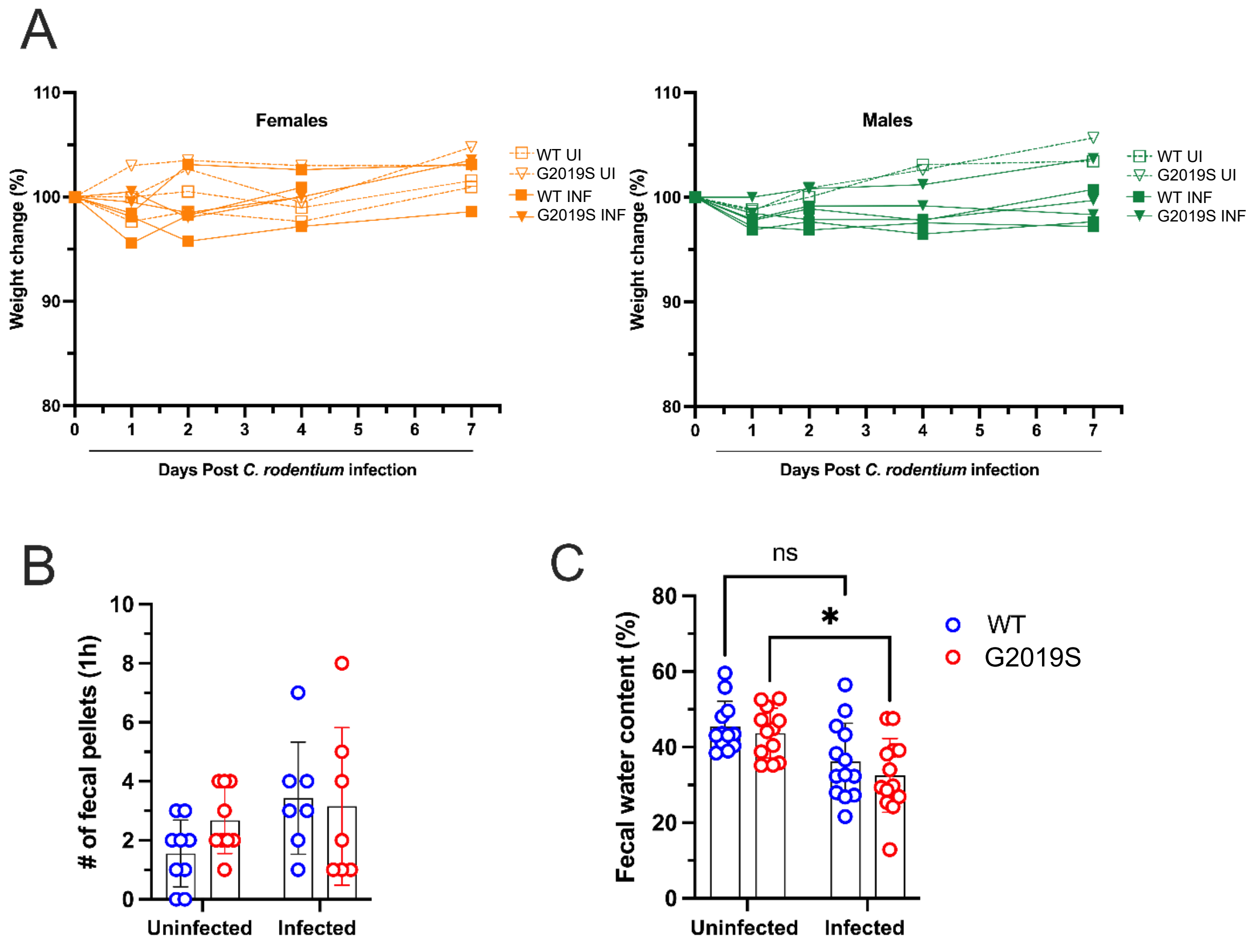

Supplemental Figure 1. *Lrrk2* G2019S mice have similar control of *C. rodentium* infection with minor changes in fecal water composition. Male and female *Lrrk2* G2019S and WT mice were infected once with approximately $1x10^9$ CFU of *C. rodentium*. (A) From days 0-7 of infection, weight change was measured. Data are represented as individual mouse points separated by sex. Depicted is one representative of two independent experiments. n=5-6 mice per group (male-to-female ratio = 0.89). On day 5 or 7 of infection, mice were placed individually in cages without bedding or food for quantification of the number of stools excreted by each animal and their water content. (B) Number of fecal pellets per hour on day 5 p.i. Data are represented as mean ± SD. Depicted is one representative graph of two independent experiments. n=6-9 mice per group. (male-to-female ratio = 0.56). (C) Water content of feces on day 7 p.i. Data are represented as mean ± SD and analyzed by two-way ANOVA with Sidak post-test. *p<0.05. Two independent experiments are presented as a pool, n=12-13 mice per group (male-to-female ratio = 0.76).

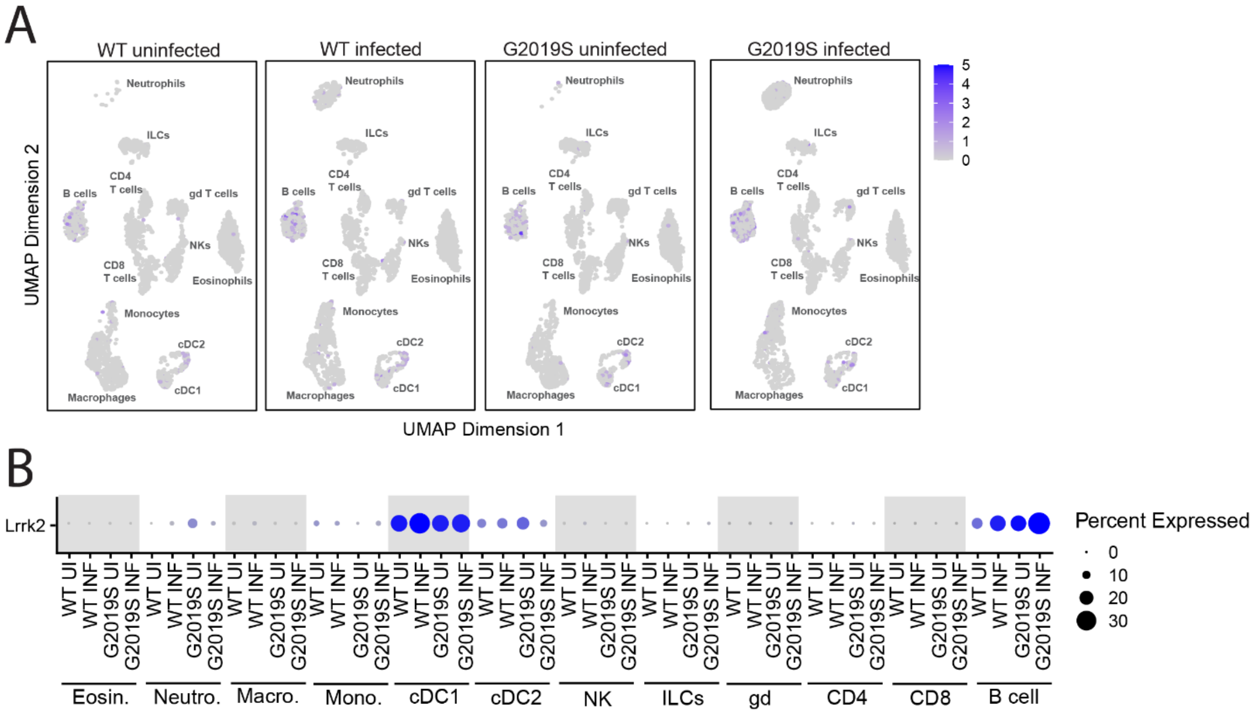

Supplemental Figure 2. *Lrrk2* expression in immunocyte populations. Male and female *Lrrk2* G2019*S* and WT mice (male-to-female ratio = 0.5) were gavaged once with approximately 1x10^9 CFUs of *C. rodentium* and colons were harvested. Immune cells were isolated for scRNAseq. (A) Feature plot of *Lrrk2* expression projected onto UMAPs, grouped by genotype and condition. (B) Dotplot depicting percent of immunocytes expressing *Lrrk2* separated by cell type, genotype, and condition. n=3 mice per group. One experiment is presented.

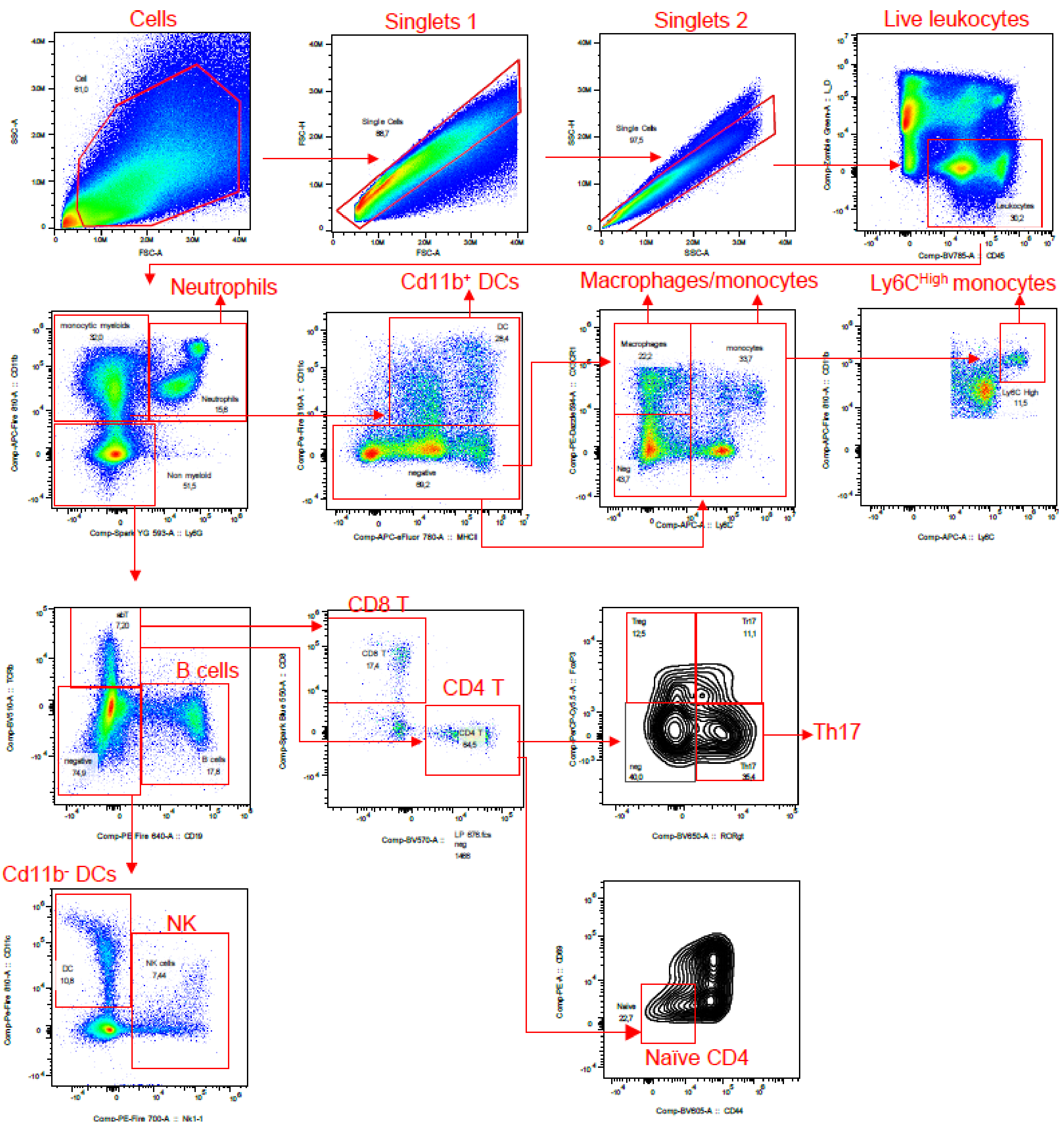

Supplemental Figure 3. Gating strategy for flow cytometry. Male and female *Lrrk2* G2019S and WT mice (male-to-female ratio = 0.9) were gavaged once with approximately $1x10^9$ CFUs of *C. rodentium* and colons were harvested. Immune cells from the colonic lamina propria were isolated for flow cytometry. Cells were stained with either surface markers or transcription factors as described in the methods. Samples and their respective controls were acquired using a spectral cytometer Aurora 4L (Cytek). Flow Cytometry Standard (FCS) files were exported and analyzed using FlowJo Software v10.9 (BD). n=5-6 mice per group. One representative of two independent experiments is shown.

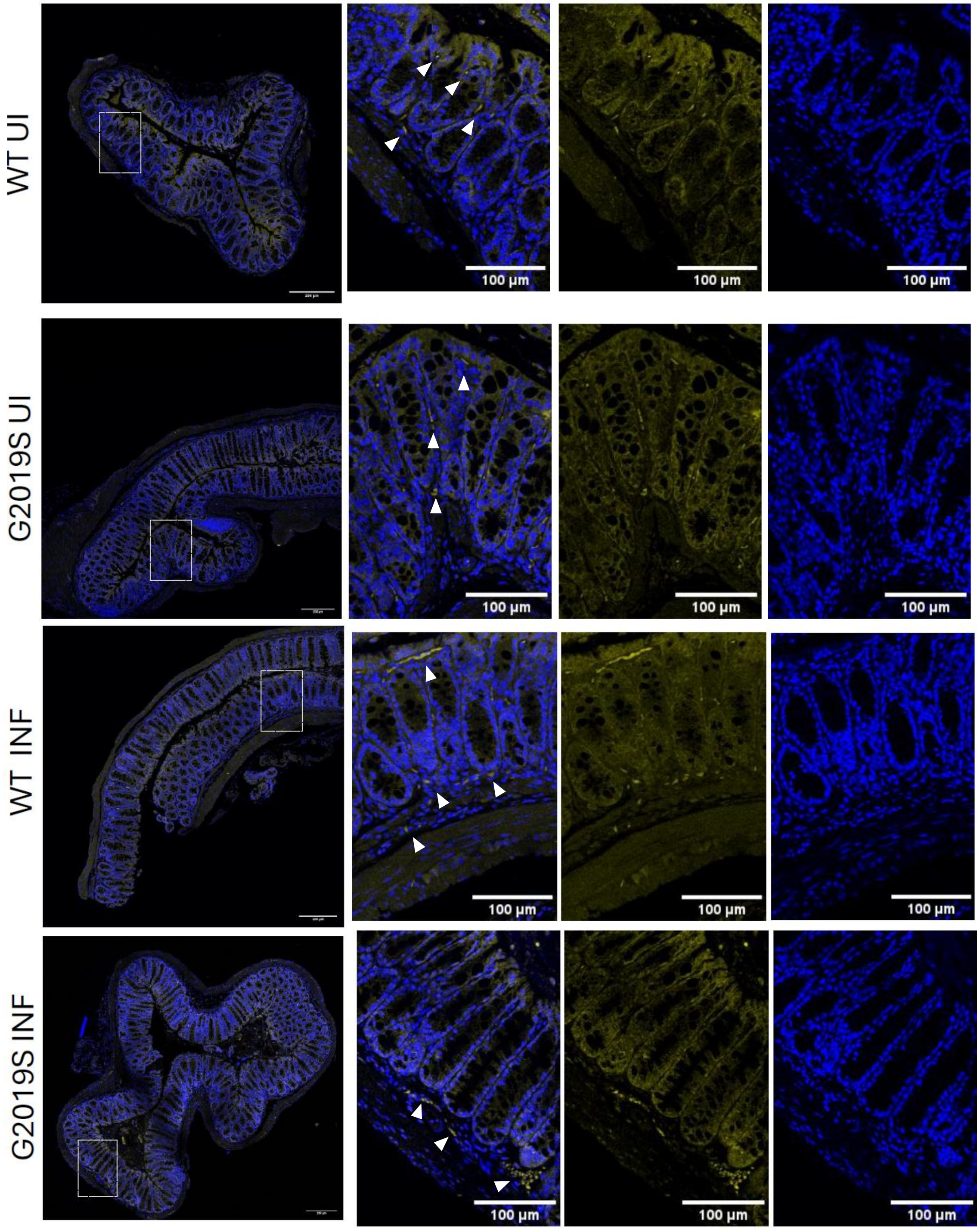

Supplemental Figure 4. Neutrophil staining is more evident at the intestinal crypt base in colons of *Lrrk2* G2019S mice after *C. rodentium* infection. Male and female *Lrrk2* G2019S and WT mice (male-to-female ratio = 0.43) were infected once with approximately $1x10^9$ CFU of *C. rodentium*. Colons were harvested 7 days after infection, fixed in 10% formalin for 24 h, washed in 70% ethanol, and diaphanized in xylene before embedding in paraffin. Sections were stained with Ly6G-AF594 antibody and DAPI, then visualized in Zeiss AXIO confocal microscope. (Left to right) Representative images are presented, followed by insets with merged staining and single channels (Ly6G – yellow, DAPI – blue). Neutrophils are highlighted with white arrows. One experiment is presented. Scale bar = 100 µm.

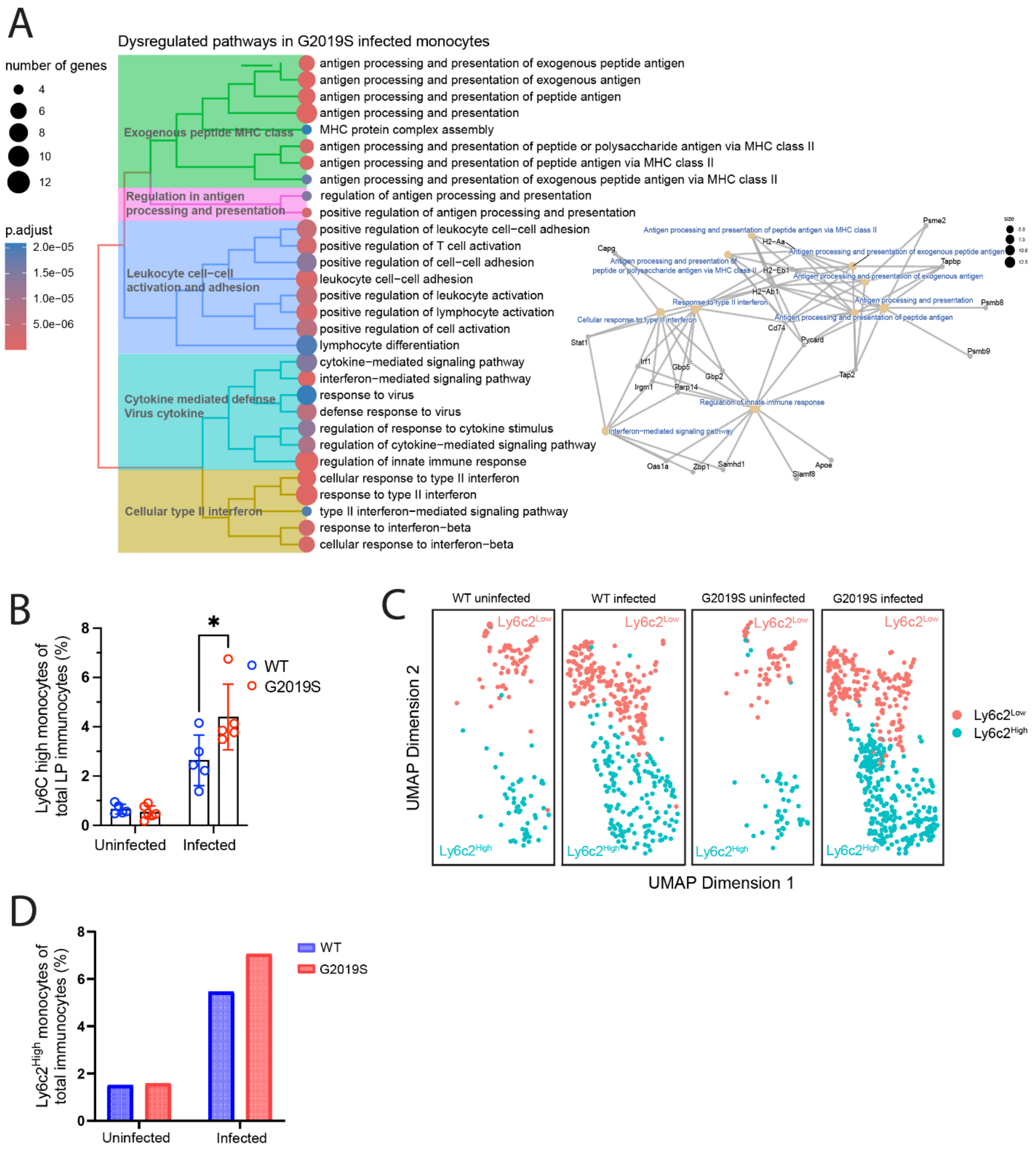

Supplemental Figure 5. ***Lrrk2*** **G2019S infected mice have increased presence of Ly6c^{high} monocytes with dysregulated differentially expressed gene profiles.** Male and female *Lrrk2* G2019S and WT mice were gavaged once with approximately 1x10^{9} CFUs of *C. rodentium* and colons were harvested. Immune cells were isolated for scRNAseq. (A) Pathway enrichment analysis completed on DEGs obtained by comparing G2019S and WT infected monocytes. DEGs were run against Gene ontology (GO) biological pathways database using *ClusterProfiler* V4.12.6 (RRID:SCR_016884). P value cut off = 0.05, q value cut off = 0.2. Top 30 GO term categories are depicted as a Treeplot (Left). Cnetplots depict association of differentially expressed gene hits, and which GO term pathways they correspond to (Right). scRNAseq was obtained from pooled conditions of n = 3 mice per group (male-to-female ratio = 0.5). One experiment is presented. (B) Percentage of Ly6c^{High} monocytes of total lamina propria immunocytes assessed through flow cytometry. Data are presented as mean ± SD and analyzed by two-way ANOVA with Sidak post-test. * $p < 0.05$. n=5-6 mice per group (male-to-female ratio = 0.9). One representative of two independent experiments is presented. (C) Subclustering of monocytes annotated as Ly6c2^{High} and Ly6c2^{Low}, depicted in a UMAP. (D) Index indicating percent of Ly6c2^{High} monocytes over total immunocytes.

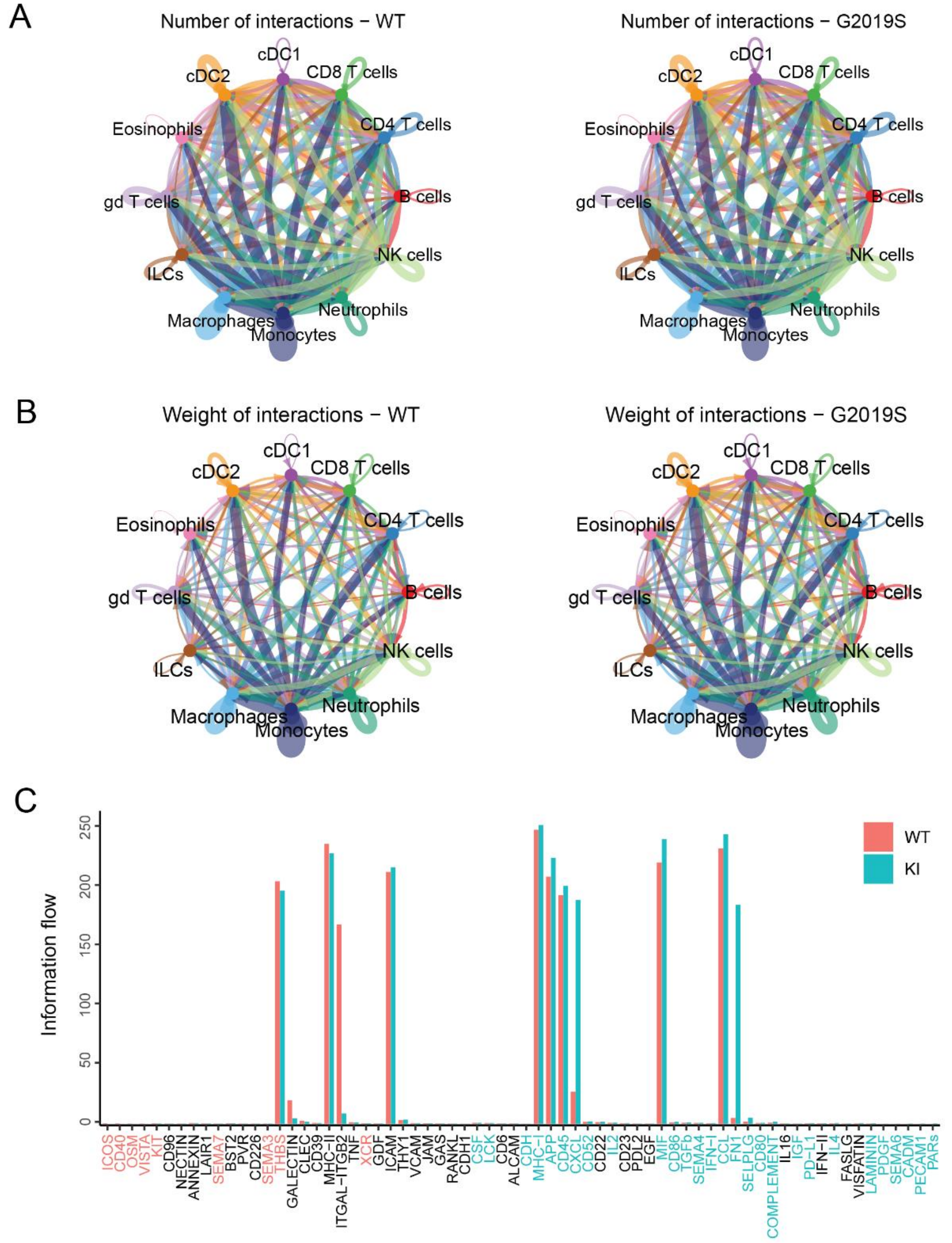

Supplemental Figure 6. CellChat analysis reveals several dysregulated signaling pathways in *Lrrk2* G2019S infected mice compared to WT. Male and female *Lrrk2* G2019S and WT mice (male-to-female ratio = 0.5) were gavaged once with approximately $1x10^9$ CFUs of *C. rodentium* and colons were harvested. Immune cells were isolated for scRNAseq. Comparative analysis between WT and G2019S infected conditions using CellChat v1.6.1 (RRID:SCR_021946) evaluating **(A)** net total interactions between all cell types or **(B)** net strength of interactions. **(C)** Comparison of dominant signaling pathways between WT and G2019S infected conditions. Information flow is calculated by the summation of ligand-receptor gene expression of one specific pathway across all pairs of sender-receiver cells. Red words indicate significant pathway enrichment in WT, blue words indicate significant pathway enrichment in G2019S, and black words indicate no clear enrichment. One experiment is presented.

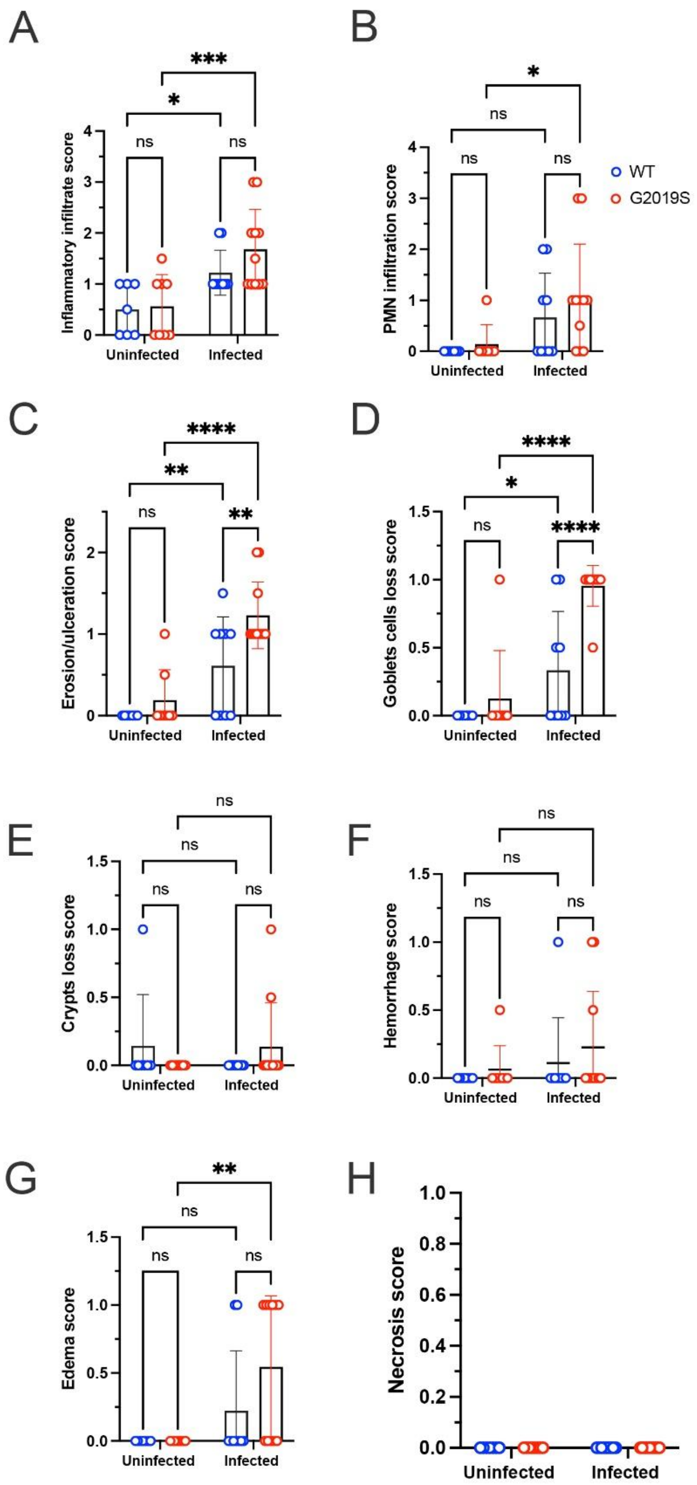

Supplemental Figure 7. Colon morphological damage score is affected by *Lrrk2* G2019S mutation after *C. rodentium* infection. Male and female *Lrrk2* G2019S and WT mice (male-to-female ratio = 0.43) were infected once with approximately 1x10^9 CFU of *C. rodentium*. Colons were harvested 7 days after infection, fixed in 10% formalin for 24 h, washed in 70% ethanol, and diaphanized in xylene before embedding in paraffin. Sections were stained with Hematoxylin and Eosin (H&E) and scored for histopathology. Histopathological scoring was conducted blindly by an expert veterinary pathologist based on the scoring criteria of colon lesions: inflammatory infiltrate (0-4), polymorphonuclear (PMN) cell infiltrate (0-4), loss of crypts (0-2), proportional loss of goblet cells (0-2), edema (0-1), erosion or ulceration (0-3), hemorrhage (0-2), and necrosis (0-1). Total pathology score (0-19) was the sum of all individual lesion scores. Data are presented as mean ± SD and analyzed by two-way ANOVA with Fisher's LSD post-test. * $p < 0.05$, ** $p < 0.01$, *** $p < 0.001$, ****$p < 0.0001$. n= 7-11 mice per group. Two independent experiments are presented as a pool.

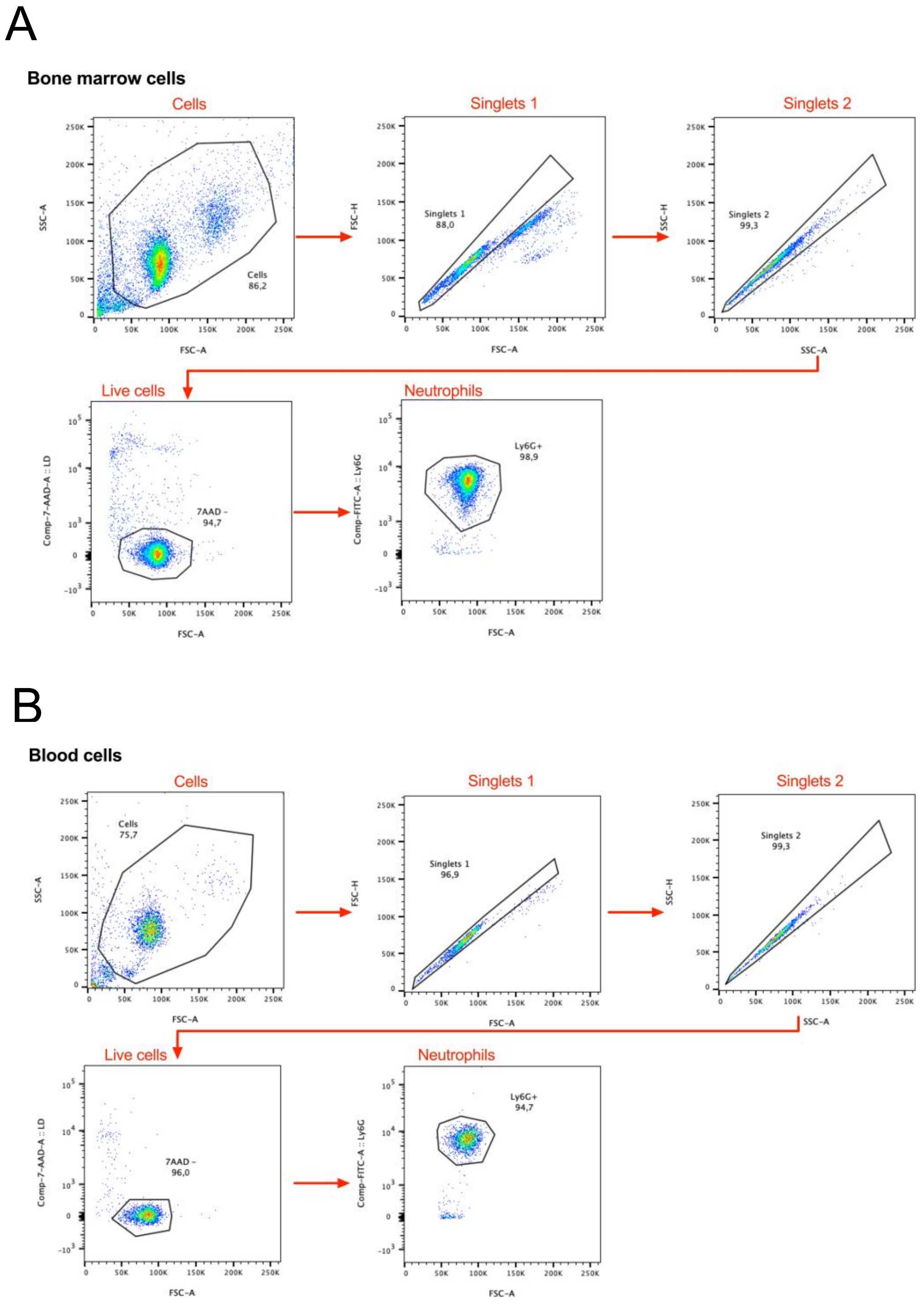

Supplemental Figure 8. Gating strategy of neutrophil purity. Whole blood, femurs, tibias, and humeri were collected from male and female *Lrrk2* G2019S and WT mice (male-to-female ratio = 0.55). Isolated neutrophils were stained for Ly6G and viability, and flow cytometry was performed. Gating strategy for neutrophil purity is presented for (A) bone marrow-isolated cells and (B) blood-isolated cells. n=3-5 mice per group. One representative of two independent experiments is shown.

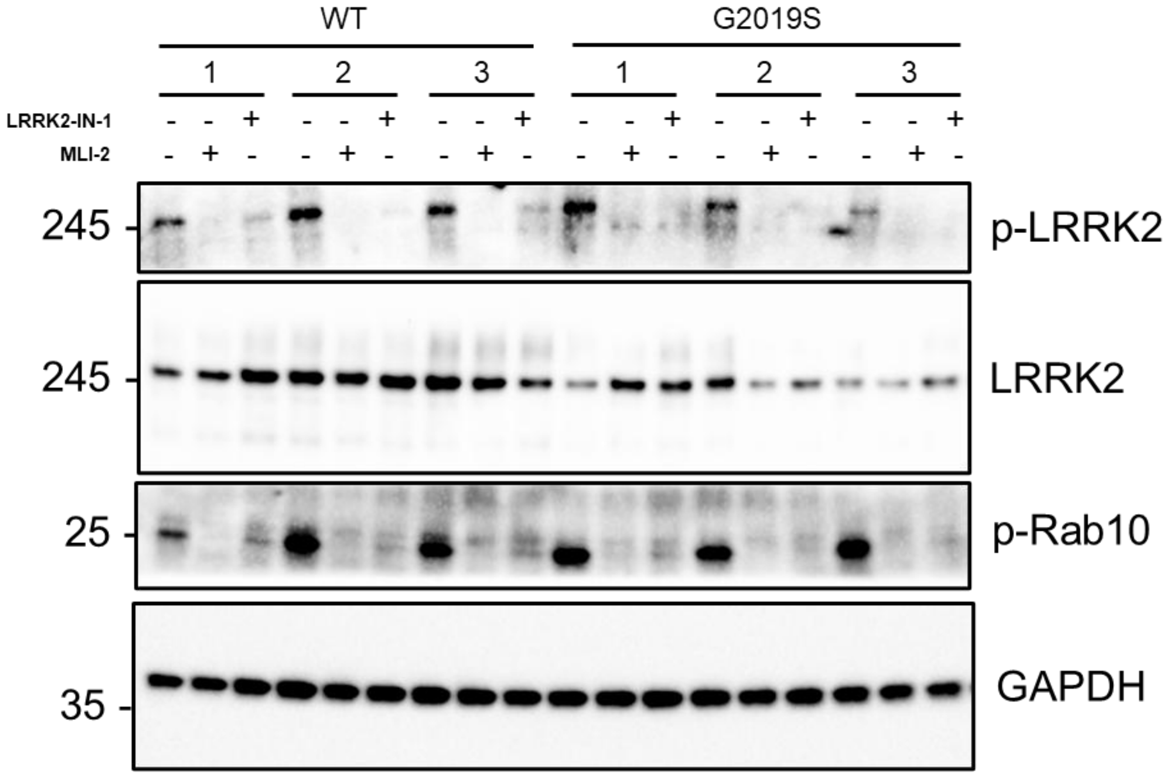

Supplemental Figure 9. Treatment with inhibitors LRRK2-IN-1 and MLi-2 reduces p-LRRK2 and p-Rab10 protein levels in bone marrow neutrophils. Bone marrow neutrophils were collected from male and female *Lrrk2* G2019S and WT mice (male-to-female ratio = 0.55). Ly6G-isolated neutrophils were incubated for two hours with MLi-2 (100 nM), LRRK2in1 (1 μM), or DMSO (vehicle control) at 37°C. Cell lysates were analyzed with immunoblotting for quantification of p-LRRK2, LRRK2, and p-Rab10 over the GAPDH housekeeping protein signal for equal loading. n=3 mice per group. Shown are representative Western blot images. One experiment is shown.

Supplemental tables

Supplemental Table 1. Top upregulated differentially expressed genes in G2019S infected monocytes compared to WT infected.

Gene	P value	Average Log2FC	Percent 1	Perfect 2	Adjusted p value
Tspo	1.5544E-18	0.57124731	0.968	0.88	3.2341E-14
Actb	8.3574E-17	0.35452497	1	1	1.7388E-12
Pkm	1.7548E-12	0.46333982	0.945	0.926	3.651E-08
Gbp2	1.9523E-12	0.92096742	0.749	0.609	4.0619E-08
Psme2	2.6937E-12	0.53264354	0.872	0.814	5.6046E-08
Zbp1	3.5213E-12	0.45609485	0.771	0.546	7.3263E-08
Psmb8	3.7592E-12	0.42689643	0.945	0.899	7.8214E-08
Prdx1	5.1239E-12	0.68532839	0.86	0.746	1.0661E-07
Eif5a	6.9447E-12	0.44724517	0.899	0.784	1.4449E-07
Msn	1.0578E-11	0.3805289	0.957	0.899	2.2008E-07
Samhd1	1.1842E-10	0.48747285	0.953	0.91	2.4638E-06
Nampt	4.9829E-10	0.61386265	0.763	0.601	1.0367E-05
Psma7	1.4332E-09	0.37404137	0.939	0.91	2.982E-05
AA467197	3.2975E-09	1.42360958	0.37	0.205	6.8609E-05
Sell	3.7509E-09	0.53296454	0.33	0.158	7.8041E-05
Stat1	4.44E-09	0.47531051	0.798	0.675	9.2379E-05
Cxcl9	8.1344E-09	1.05573072	0.37	0.194	0.00016924
Pomp	9.234E-09	0.31834424	0.901	0.869	0.00019212
Tapbp	1.1988E-08	0.4351979	0.753	0.658	0.00024943
Irf1	1.4011E-08	0.58549975	0.745	0.623	0.00029151
Irgm1	2.5417E-08	0.45803956	0.403	0.227	0.00052882
Bst1	2.5795E-08	0.42046847	0.455	0.292	0.0005367
Gbp5	2.8314E-08	0.64189455	0.561	0.388	0.0005891
Tap2	2.9721E-08	0.4067505	0.516	0.361	0.00061838
Capg	3.3778E-08	0.41847943	0.775	0.645	0.00070278
Ms4a6d	8.5524E-08	0.50867688	0.814	0.749	0.00177941
Oas1a	9.7654E-08	0.31920261	0.294	0.145	0.00203178
Pnp	1.6871E-07	0.34274729	0.745	0.626	0.00351013
Slamf8	2.3142E-07	0.4046183	0.496	0.336	0.00481488
Parp14	2.7597E-07	0.43140425	0.621	0.489	0.00574188
Anxa2	3.189E-07	0.39891524	0.903	0.855	0.00663506
Ostf1	3.3035E-07	0.3100454	0.816	0.754	0.00687323
Psmb9	6.223E-07	0.35397462	0.771	0.683	0.01294758
Pfn1	7.4076E-07	0.25317366	0.994	0.978	0.01541226
Txn1	7.501E-07	0.38181141	0.949	0.91	0.01560661
Ifi47	8.9318E-07	0.40353798	0.383	0.23	0.01858357
Chil3	9.2842E-07	0.80522385	0.472	0.333	0.01931672
Fam26f	9.906E-07	0.71935509	0.46	0.317	0.02061048
Cnn3	9.9947E-07	0.46080539	0.259	0.134	0.02079492
Cycs	1.1E-06	0.30458111	0.727	0.626	0.02288581
Sdcbp	1.3885E-06	0.28880599	0.953	0.921	0.02888974

Ly6c2	1.4256E-06	0.48211585	0.652	0.503	0.02966033
Ralgds	1.4768E-06	0.36427652	0.362	0.23	0.03072726
Sppl2a	1.5688E-06	0.32621139	0.747	0.65	0.03264094
Pgk1	1.8181E-06	0.40699509	0.779	0.691	0.03782704
Vcan	1.8654E-06	0.6711977	0.415	0.281	0.03881184
Prdx5	2.0805E-06	0.47424389	0.968	0.956	0.04328785
Pycard	2.2865E-06	0.34949264	0.757	0.672	0.04757273
Atp5g1	2.3364E-06	0.26721565	0.783	0.669	0.04861076

Supplemental table 2. Top downregulated differentially expressed genes in G2019S infected monocytes compared to WT infected.

Gene	P value	Average Log2FC	Percent 1	Percect 2	Adjusted p value
Rpl41	1.7064E-26	-0.3450237	1	0.997	3.5503E-22
Igha	3.0077E-20	-0.4620335	0.994	1	6.2578E-16
Rpl35a	8.9664E-18	-0.2546594	1	1	1.8656E-13
Gm34084	1.4978E-14	-0.6716632	0.271	0.511	3.1163E-10
H2-Aa	1.8112E-14	-0.7555772	0.761	0.877	3.7684E-10
Cd74	1.045E-12	-0.573703	0.901	0.962	2.1743E-08
H2-Ab1	1.6319E-12	-0.653013	0.773	0.877	3.3953E-08
Ighg1	2.1989E-12	-0.3647538	0.087	0.268	4.575E-08
Rplp1	6.6052E-12	-0.25635	1	1	1.3743E-07
Rpl37	1.5241E-11	-0.2552487	0.998	1	3.171E-07
H2-Eb1	2.9593E-11	-0.6496714	0.692	0.833	6.1572E-07
Rps27rt	5.4651E-11	-0.3716211	0.253	0.473	1.1371E-06
Igkc	9.8345E-11	-0.27532	0.998	0.997	2.0462E-06
Selenop	1.9127E-10	-0.6048694	0.623	0.768	3.9796E-06
Ptpn18	1.3309E-09	-0.3725759	0.787	0.915	2.769E-05
Malat1	1.4626E-09	-0.2720217	1	1	3.0432E-05
Apoe	3.4149E-09	-0.687838	0.919	0.915	7.105E-05
Rps26	5.1835E-09	-0.2541757	0.994	0.997	0.00010785
Fosb	1.6286E-08	-0.6431246	0.632	0.76	0.00033886
Rhog	8.8776E-08	-0.3651707	0.721	0.828	0.00184707
Rgs10	1.0016E-07	-0.3914292	0.472	0.631	0.0020839
Rel	1.2101E-06	-0.3216912	0.931	0.967	0.02517838
Iqgap2	1.4678E-06	-0.2714566	0.188	0.342	0.03053922
Zfp36l1	1.8925E-06	-0.4646346	0.583	0.757	0.03937459
